# Crystallography relevant to Mars and Galilean icy moons: crystal behavior of kieserite-type monohydrate sulfates at extraterrestrial conditions down to 15 K

**DOI:** 10.1107/S2052252521012720

**Published:** 2022-01-11

**Authors:** Manfred Wildner, Boris A. Zakharov, Nikita E. Bogdanov, Dominik Talla, Elena V. Boldyreva, Ronald Miletich

**Affiliations:** a Department of Mineralogy and Crystallography, University of Vienna, Althanstraße 14, A-1090 Wien, Austria; b Boreskov Institute of Catalysis, Siberian Branch of the Russian Academy of Sciences, Lavrentieva Avenue 5, Novosibirsk 630090, Russian Federation; c Novosibirsk State University, Pirogova Street 2, Novosibirsk 630090, Russian Federation

**Keywords:** monohydrate sulfate kieserites, Galilean icy moons, He cryojet techniques

## Abstract

Monohydrate sulfate kieserites (*M* ^2+^SO_4_·H_2_O) and their solid solutions are essential constituents on the surface of Mars and most likely also on Galilean icy moons in our solar system. Phase stabilities of end-member representatives (*M* ^2+^ = Mg, Fe, Co, Ni) have been examined crystallographically using single-crystal X-ray diffraction at 1 bar and temperatures down to 15 K, by means of applying open He cryojet techniques at in-house laboratory instrumentation. All four representative phases show a comparable, highly anisotropic thermal expansion behavior with a remarkable negative thermal expansion along the monoclinic *b* axis and a pronounced anisotropic expansion perpendicular to it. The lattice changes down to 15 K correspond to an ‘inverse thermal pressure’ of approximately 0.7 GPa, which is far below the critical pressures of transition under hydro­static compression (*P_c_* ≥ 2.40 GPa). Consequently, no equivalent structural phase transition was observed for any compound, and neither dehydration nor rearrangements of the hydrogen bonding schemes have been observed. The *M* ^2+^SO_4_·H_2_O (*M* ^2+^ = Mg, Fe, Co, Ni) end-member phases preserve the kieserite-type *C*2/*c* symmetry; hydrogen bonds and other structural details were found to vary smoothly down to the lowest experimental temperature. These findings serve as an important basis for the assignment of sulfate-related signals in remote-sensing data obtained from orbiters at celestial bodies, as well as for thermodynamic considerations and modeling of properties of kieserite-type sulfate monohydrates relevant to extraterrestrial sulfate associations at very low temperatures.

## Abstract 2

The low-temperature crystallography of kieserite-type monohydrate sulfates, relevant for Mars and Galilean icy moons, has been investigated down to 15 K, revealing a pronounced anisotropy of their thermal expansion behavior. The present results provide a reliable basis for the proper assignment of sulfate-related signals obtained from remote-sensing data from orbiters at celestial bodies.

## 1. Introduction

The abundance of sulfate minerals, in particular hydrated magnesium sulfates (MgSO_4_·*n*H_2_O), has been confirmed on Mars and elsewhere in the solar system starting with the first *in situ* discovery in martian soils by the Viking Mars probe (Keil *et al.*, 1978 ▸; Clark & Van Hart, 1981 ▸). The ability of these materials to release or absorb water and the prevailing humidity, even forming liquid brines (Peterson & Wang, 2006 ▸), ranks them among critical components governing the water cycles on the surface of Mars and other celestial bodies. The influence of temperature, humidity and pressure on dehydration and rehydration capabilities has been investigated in various laboratory studies (*e.g.* Wang *et al.*, 2009 ▸; Nakamura & Ohtani, 2011 ▸; Fortes *et al.*, 2017*a* ▸). Among these MgSO_4_·*n*H_2_O phases, covering a wide range of hydration states (*n*) from 0 to 11, the monohydrate sulfate kieserite and its solid solutions with transition metal (TM) counterparts (Papike *et al.*, 2007 ▸) dominate the deposits at lower martian latitudes, where it is assumed to be an essential H_2_O carrier (*e.g.* Christensen *et al.*, 2004 ▸; Arvidson *et al.*, 2005 ▸; Clark *et al.*, 2005 ▸; Bishop *et al.*, 2009 ▸).

However, there is some ongoing dispute regarding the stability of kieserite on the martian surface. On the one hand, Chipera & Vaniman (2007 ▸) argue that the relative humidity should lead to its rehydration to starkeyite, MgSO_4_·4H_2_O, which is thermodynamically stable under the prevailing conditions. On the other hand, Wang *et al.* (2009 ▸) found that the presence of Ca- and Fe-sulfates and Fe-oxides or Fe-hydroxides enhances the dehydration of higher Mg-sulfates down to the monohydrate. Smectite minerals were shown to have a stabilizing effect on the hydration states, allowing lower sulfate hydrates to exist well outside their stability range (Wilson & Bish, 2012 ▸). Furthermore, there is evidence that martian kieserite actually has an intermediate composition between the end members kieserite, MgSO_4_·H_2_O, and szomolnokite, FeSO_4_·H_2_O (Bishop *et al.*, 2009 ▸). The respective synthetic materials used for the investigation of the given solid solution (Talla & Wildner, 2019 ▸) show no sign of rehydration even after several years under ambient conditions as soon as the Fe content exceeds 0.2 apfu.

A related debate concerns the existence and nature of a postulated second structural polytype of kieserite with a broader stability range but similar spectroscopic properties, the so-called low-humidity (LH) kieserite, presumed by several authors to prevail on Mars (Wang *et al.*, 2009 ▸; Jamieson *et al.*, 2014 ▸). While elucidation of the actual structure and properties of this enigmatic ‘LH phase’ is still pending to date, it is one of the goals of the present study to provide reliable – hitherto missing – low-temperature reference data of ‘classical’ *M*SO_4_·H_2_O compounds, to serve as a benchmark for comparison and as a basis for spectroscopic and thermodynamic modeling purposes.

Spectroscopic data recorded by orbiters reveal sulfate-rich regions to be present not only on the martian surface, but also appear to be an abundant feature on many other celestial bodies in our solar system, *i.e.* on the icy moons of Jupiter and Saturn (Kargel, 1991 ▸; McCord *et al.*, 2001*a* ▸). Higher hydrated Mg-sulfates (*n* = 6–11) are assumed to be relevant non-ice mineral constituents on the surface but also in the ice mantles of the Galilean moons (*e.g.* McCord *et al.*, 2001*a* ▸; Dalton *et al.*, 2005 ▸, 2012 ▸). While McCord *et al.* (2001*b* ▸) did not find any indication of Mg-sulfate radiolysis when subjecting epsomite (*n* = 7) to a 100 eV electron beam, Tani *et al.* (2012 ▸) determined the formation of hydrogen and sulfite radicals at 90 K by exposure to gamma radiation. More importantly, several studies have shown that the interaction of UV or other energetic radiation with highly hydrated sulfates significantly catalyzes and speeds up their dehydration. Under such influence, epsomite is converted to hexahydrite [the mineral name for the hexahydrate, MgSO_4_·6H_2_O (Cardell *et al.*, 2007 ▸)], the further dehydration of which has also been proven to be catalyzed by UV radiation (Cloutis *et al.*, 2007 ▸). This processes could lead to the formation of sulfate monohydrates on the icy surface of the Galilean moons.

Moreover, lower-hydrated or even anhydrous sulfates are also expected to be present within the deeper mantle and core of these planetary bodies (Kargel, 1991 ▸; Nakamura & Ohtani, 2011 ▸; Meusburger *et al.*, 2020 ▸). Depending on their thermodynamic stability, they play a key role for the formation of subsurface oceans, which could even contain extraterrestrial life, as discussed for the south-polar region of Saturn’s moon Enceladus, where a subsurface body of liquid exists due to tidal heating (Solomonidou *et al.*, 2011 ▸). However, no clear confirmation of the presence of sulfates on this latter object or in its hydro­thermal plumes exists, in part because of significant overlaps of rather broad bands and combination modes of various sulfate minerals in the standard <5 µm spectral range covered by orbiters (Bishop *et al.*, 2004 ▸). This problem becomes even more remarkable when the deposits are spatially restricted with other phases present, necessitating the use of complex spectral unmixing models (*e.g.* Combe *et al.*, 2008 ▸). However, it is most probable that the rocky core of the moon corresponds to C1/C2 chondritic composition (Kargel, 1991 ▸; Sekine *et al.*, 2015 ▸), where high sulfate contents are present in the soluble fraction (Fredriksson & Kerridge, 1988 ▸; Burgess *et al.*, 1991 ▸), filling abundant brecciation cracks (Richardson, 1978 ▸); therefore the presence of sulfates also on Enceladus will probably be confirmed by future missions.

Following our investigations on the structural crystallography of kieserite-type solid solutions: *M* ^2+^SO_4_·H_2_O (*M* ^2+^ = Mg, Fe, Co, Ni), including the temperature-dependent behavior of the end members at temperatures prevailing at equatorial martian latitudes (Bechtold & Wildner, 2016 ▸; Talla & Wildner, 2019 ▸; Talla *et al.*, 2020 ▸), we recently carried out investigations under high-pressure conditions relevant to the interior of individual icy moons (Meusburger *et al.*, 2019 ▸, 2020 ▸; Ende *et al.*, 2020 ▸; Wildner *et al.*, 2021 ▸). These experimental studies revealed a second-order phase transition from the monoclinic α-phase (space group *C*2/*c*) to a triclinic β-phase (space group *P* 1) at critical transition pressures ranging from 2.40 (Co) to 6.15 GPa (Fe). The findings from high-pressure crystallography show a partial rearrangement of the hydrogen-bonding scheme as the most obvious structural change. Earlier studies down to 110 K (Talla & Wildner, 2019 ▸, Talla *et al.*, 2020 ▸) gave no evidence of a comparable transition, however, it can not be ruled out to occur at temperatures even lower than the previously covered range. Although on Mars the temperatures in equatorial latitudes range between 280 and 170 K (Witzke *et al.*, 2007 ▸), significantly lower temperatures can be expected on the surface of the icy satellites. The temperature changes during the seasonal and diurnal cycles on some of the icy moons of Jupiter and Saturn have been modeled, such as for Europa (Ashkenazy, 2019 ▸), where the mean annual temperature range spans (depending on the assumed internal heating rate) from 94 to 98 K in equatorial latitudes and from 35 to 62 K on the poles. The measurements during the Rosetta mission to comet 67P showed evidence of SO_2_ in its plume and ammonium sulfate on its surface, suggesting the presence of sulfate on cometary bodies and asteroids (Poch *et al.*, 2020 ▸). In such cases, they are exposed to temperatures as low as those of the cosmic vacuum, amounting to a mere 2.7 K.

Since the reliable knowledge of phase relations and structural details of relevant sulfate minerals at astrophysically significant conditions is of utmost importance for their identification and discrimination in remote-sensing spectroscopic data from orbiters, we extend our investigations on end-member monohydrate sulfates well down to temperatures as low as 15 K. To reveal the structural changes, *in situ* low-temperature X-ray crystallography was carried out at temperature conditions relevant to the icy moons of Jupiter and Saturn and comparable objects in the outer solar system, as well as the polar regions of Mars or dwarf planets and asteroids within the inner solar system. At the same time, this study aims to verify any potential structural phase transition equivalent to those reported earlier under compression of kieserite-type phases, following the analogy of ‘inverse thermal pressure’ on cooling.

## 2. Experimental

### 2.1. Temperature-dependent single-crystal X-ray data collection

Single crystals of synthetic kieserite-type compounds *M* ^2+^SO_4_·H_2_O (*M* ^2+^ = Mg, Fe, Co, Ni) for the present data collections were extracted from the respective end-member batches prepared earlier (Bechtold & Wildner, 2016 ▸; Talla & Wildner, 2019 ▸; Talla *et al.*, 2020 ▸). Data collections between 313 and 113 K were performed in steps of 40 K on a Bruker Apex II diffractometer equipped with a CCD area detector and an Incoatec Microfocus Source IµS (30 W, multilayer mirror, Mo *K*α), in a dry stream of nitro­gen (Cryostream 800, Oxford Cryosystems). Several sets of φ- and ω-scans with a 2° scan width were measured at a crystal–detector distance of 40 mm up to 80° 2θ full sphere. Absorption was corrected by evaluation of multi-scans. Measurements and correction procedures were performed with the *SAINT* software package (Bruker AXS, 2012 ▸). Data collections between 75 and 15 K (and at room temperature) were performed in steps of 15 K with Mo *K*α radiation on an Oxford Diffraction Gemini R Ultra diffractometer with a CCD detector. The open-flow helium cryostat Helijet from Oxford Diffraction with helium coaxial flow shielding was used to maintain low temperatures of the samples. Data collections and treatments were performed using *CrysAlisPro* software (Rigaku Oxford Diffraction, 2016 ▸). During several of the scheduled measurements we encountered a problem of excessive formation of an ’ice’-cover on the sample crystals, composed from solidified air components, mainly O_2_ and N_2_, in the open-flow helium cryostat, leading to interference of sample reflections with the emergent multi-grain powder rings (see Zakharov *et al.*, 2021 ▸). These datasets were excluded from consideration: respective data collections were aborted and/or deleted from the further analyses. Data collection was resumed after the problem was fixed or, at least, partly solved by changing the crystal holder type and other preventive measures.

### 2.2. Crystal structure refinements

All structure refinements were performed on *F* ^2^ with *SHELXL* (Sheldrick, 2015 ▸) in the ‘traditional’ non-reduced kieserite cell setting (*cf*. Wildner & Giester, 1991 ▸) in the space group *C*2/*c*. Scattering curves for neutral atoms were used. Note that, for the purposes of data consistency and comparability, the cell axis parameters from the temperature-dependent measurements were corrected in such a way that respective values at 293 K (interpolated for Apex II measurements) match those from earlier room-temperature (RT) studies, obtained on a Nonius Kappa CCD diffractometer (see Bechtold & Wildner, 2016 ▸; Talla & Wildner, 2019 ▸; Talla *et al.*, 2020 ▸), since the latter RT data proved to most closely match corresponding high-precision lattice parameters obtained with the 8-position centering mode (King & Finger, 1979 ▸) on a Stoe AED II diffractometer. The respective corrections were in the range ≤2‰. Further note that the structures of the Mg and Fe compounds at 193 and 113 K have already been published by Talla & Wildner (2019 ▸) and those of NiSO_4_·H_2_O at 273, 193 and 113 K by Talla *et al.* (2020 ▸). Crystal structure drawings throughout the paper were prepared with the program *VESTA3* (Momma & Izumi, 2011 ▸).

### 2.3. Thermal equation of state calculations

Due to the limited number of temperature-dependent unit-cell data points (8–10 at most) obtained from full single-crystal structure determinations, we preferred to keep the thermal equation of state (EoS) analyses as simple as possible and with a minimum of variable parameters. The unit-cell axes lengths and cell volumes between 15 and 313 K were fitted therefore to a Fei-type (Fei, 1995 ▸) thermal EoS using the *EoSFit7c* and *EoSFit7*-GUI programs by Angel and coworkers (Angel *et al.*, 2014 ▸; Gonzalez-Platas *et al.*, 2016 ▸). The reference temperature was chosen at *T*_ref_ = 293 K; α_2_ was fixed at 0 (see also Angel *et al.*, 2014 ▸), *i.e.* simplifying the thermal EoS to the linear approach with two coefficients following α = α_0_ + α_1_ *T*. Strain and thermal expansion tensors were calculated from the thermal EoS with the Strain utility in the *EoSFit7c* program.

## 3. Results and discussion

### 3.1. General aspects

**Figure 1:**
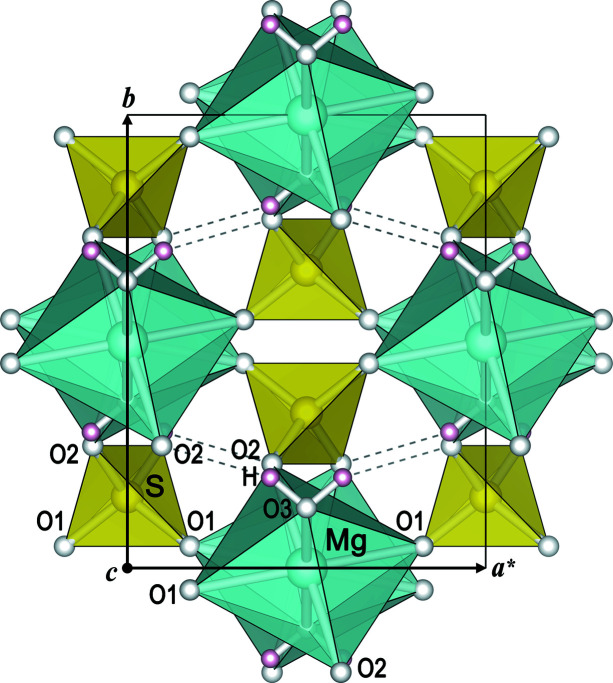
Crystal structure of kieserite (MgSO_4_·H_2_O) at 15 K, projected along the *c* axis, *i.e.* along the octahedral–tetrahedral chains.

Selected crystal data and details of the data collections and structure refinements are summarized in Table S1 of the supporting information. Final atomic parameters are listed in Table S2, and results of the thermal EoS and strain calculations in Table 1 ▸. The most relevant interatomic distances and angles are given in Table S3. Under ambient conditions, kieserite and isotypic compounds *M* ^2+^SO_4_·H_2_O (*M* ^2+^ = Mg, Fe, Co, Ni) crystallize with monoclinic symmetry in the space group *C*2/*c* (Hawthorne *et al.*, 1987 ▸; Wildner & Giester, 1991 ▸). The crystal structure of the Mg-representative kieserite is illustrated in Fig. 1 ▸ projected along the crystallographic *c* axis. The structures are built from kinked chains of O3(H_2_O)-corner-sharing *M*O_4_(H_2_O)_2_ octahedra (point symmetry 1), elongated along their O3–O3 axes, and nearly regular SO_4_ tetrahedra (point symmetry 2), intra-linking adjacent octahedra within the chains by common O2 corners. These octahedral–tetrahedral chains are aligned parallel to the *c* axis (*i.e.* along the viewing direction in Fig. 1 ▸) and interlinked to a framework by sharing the remaining polyhedral O1 corners as well as by moderately strong O3–H⋯O2 hydrogen bonds.

### 3.2. Thermal expansion of the unit cell

**Figure 2:**
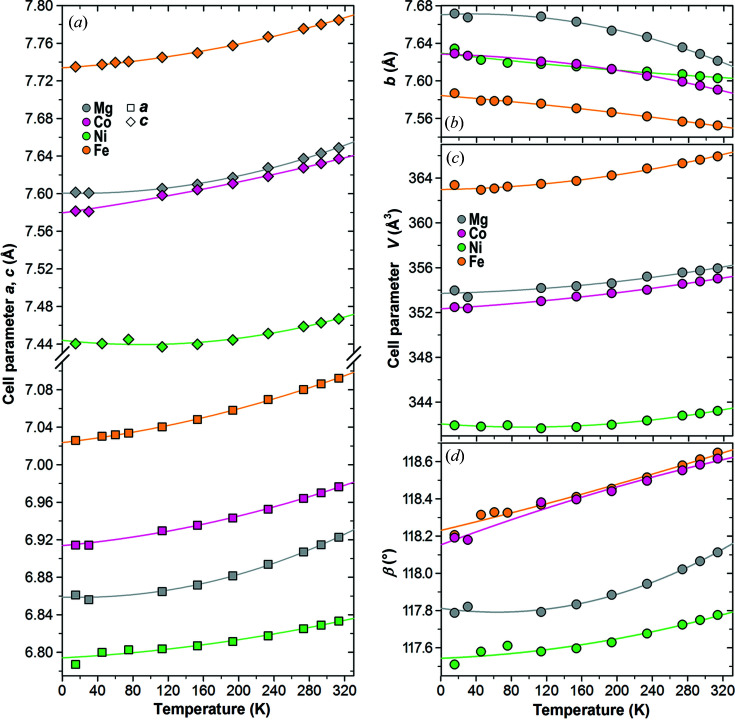
Unit-cell dimensions of kieserite-type compounds *M* ^2+^SO_4_·H_2_O (*M* ^2+^ = Mg, Fe, Co, Ni) in the temperature range 15–313 K with lines representing the Fei-type EoS results (Table 1 ▸): (*a*) *a* and *c* axes; (*b*) *b* axes; (*c*) unit-cell volumes; and (*d*) monoclinic angle β. For errors see the underlying data in Tables 1 ▸, S1 and S2.

The lattice dimensions, depicted in Fig. 2 ▸, as well as the structural properties of the kieserite-group representatives deviate more or less from a linear fashion with decreasing temperature. While approximately linear shifts of lattice parameters are usually observed in high-temperature studies from RT upwards, the tendency to flatten below around 100 K towards absolute zero is well known for many crystalline solids (*e.g.* Drebushchak, 2020 ▸), and even an inversion of the trends leading to negative bulk volume expansion may be observed below ∼50 K; respective examples among sulfates are higher hydrates like meridianiite [MgSO_4_·11H_2_O (Fortes *et al.*, 2008 ▸)] or mirabilite [Na_2_SO_4_·10H_2_O (Brand *et al.*, 2009 ▸)].

The thermal expansion parameters and Fei-type EoS coefficients of the title compounds are summarized in Table 1 ▸. As expected, the cell volumes decrease on cooling [Fig. 2 ▸(*c*)], as do the *a* and *c* axes and the monoclinic angle β [Figs. 2 ▸(*a*) and 2(*d*)], whereas the *b* axes lengthen in all four compounds over the full investigated temperature range [Fig. 2 ▸(*b*)], showing a pronounced negative thermal expansion (NTE) effect. The present NTE behavior is even more remarkable since, with α_*b*,293_ ranging between −4.4 × 10^−5^ K^−1^ in kieserite (Mg) and −0.8 × 10^−5^ K^−1^ in NiSO_4_·H_2_O, it amounts to a magnitude comparable to the positive thermal expansion of the *a* and *c* axes, and it furthermore shows no sign of turning positive or at least flattenning towards higher temperatures. NTE behavior of single lattice axes in otherwise ‘thermally normal’ compounds is not uncommon, but usually at a much lower rate. For example, in epsomite (MgSO_4_·7H_2_O) and meridianiite (MgSO_4_·11H_2_O) each one of their lattice axes shows NTE (in the former case with an already high α of at most −2 × 10^−5^ K^−1^), both turning positive at ∼250 K (Fortes *et al.*, 2006 ▸, 2008 ▸); in chalcanthite, CuSO_4_·5H_2_O, a very weak axial NTE is observed which flattens towards ∼300 K (Schofield & Knight, 2000 ▸). The strong NTE effect of the *b* axes in the title compounds is further discussed below (Section 3.4).

Regarding the bulk volume thermal expansion α_*V*_ [Table 1 ▸, Fig. 2 ▸(*c*)], the Fe compound with the largest cell volume also shows the strongest volume expansion (4.7 × 10^−5^ K^−1^), while, within limits of error, the Mg and Co compounds (similar cell volumes) but also the Ni phase with the smallest cell volume, all have the same smaller volume expansion (∼3.4 × 10^−5^ K^−1^). The flattening of the cell volumes towards low temperatures seems to be somewhat more pronounced in the Ni and Mg phases compared with those of Co and especially Fe. MgSO_4_·H_2_O consistently exhibits the strongest changes of the individual cell-edge lengths with temperature, especially concerning the excessive negative thermal expansion of the *b* axes, whereas NiSO_4_·H_2_O shows a significantly smaller average change of the axial lengths [Figs. 2 ▸(*a*) and 2(*b*)]. Among the four compounds, the *a* axes show a slightly higher expansion than the *c* axes, apart from the insignificant inversion in NiSO_4_·H_2_O (Table 1 ▸).

**Figure 3:**
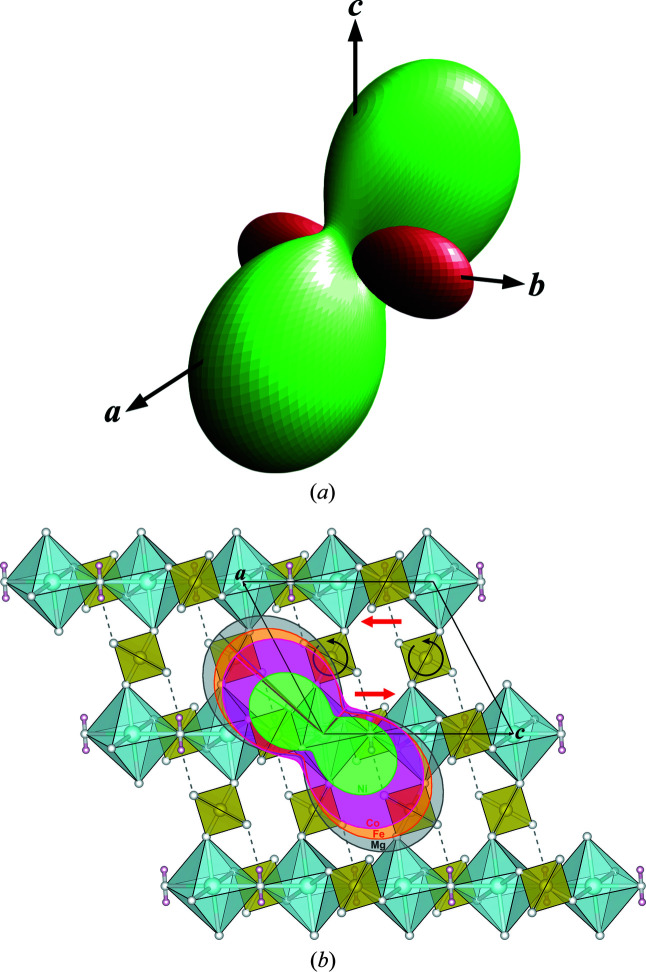
(*a*) Thermal expansion tensor in MgSO_4_·H_2_O at 113 K (*T*_ref_ = 293 K), with green indicating positive values, red indicating negative values; (*b*) cross sections of the thermal expansion tensors of kieserite-type compounds *M* ^2+^SO_4_·H_2_O (*M* ^2+^ = Mg, Fe, Co, Ni) at 113 K (*T*_ref_ = 293 K) in the *a* *c* plane, superimposed on a respective projection of the structure of kieserite at 113 K. For increasing temperature, black arrows show the sense of tetrahedral rotations around the twofold axis, red arrows indicate the resulting opposing translations of neighboring octahedral–tetrahedral chains within the *a* *c* plane (see text). The tensor surface and cross sections were prepared with the program *WinTensor* (Kaminski, 2014 ▸).

The respective thermal expansion tensors depicted in Figs. 3 ▸(*a*) and 3(*b*) (derived from the strain components in Table 1 ▸) exhibit pronounced anisotropy also in the plane perpendicular to the NTE-affected *b* axes, not evident from the more or less similar thermal expansion values α_*a*,*c*_ of the *a* and *c* axes (Table 1 ▸). As illustrated in Fig. 3 ▸(*b*), the respective smallest positive eigenvalues are oriented approximately parallel to the short cell diagonal in the *a* *c* plane, the largest ones approximately parallel to [201]. For the Ni compound, these directions deviate slightly (∼9°) from the quite consistent orientation in the other compounds. The relation between the anisotropy of thermal expansion and stereochemical changes with temperature is further discussed in Section 3.4.

The volume thermal expansion α_*V*_ of the investigated kieserite-type compounds compares well with that of anhydrous sulfates. For example, in α- and β-MgSO_4_ the values at 300 K are 3.7 × 10^−5^ and 4.1 × 10^−5^ K^−1^, respectively (Fortes *et al.*, 2007 ▸), in anhydrite (CaSO_4_) it is 3.7 × 10^−5^ K^−1^ at RT (Evans, 1979 ▸). Higher hydrated sulfates usually also exhibit larger thermal expansion coefficients at or close to RT: in gypsum [CaSO_4_·2H_2_O (Schofield *et al.*, 1996 ▸)] α_*V*_ is 7.0 × 10^−5^ K^−1^ (320 K), in epsomite [MgSO_4_·7H_2_O (Fortes *et al.*, 2006 ▸)] it is 11 × 10^−5^ K^−1^ (300 K), in MgSO_4_·9H_2_O (Fortes *et al.*, 2017*b* ▸) it is 11.3 × 10^−5^ K^−1^ (250 K), in mirabilite [Na_2_SO_4_·10H_2_O (Brand *et al.*, 2009 ▸)] it is 11 × 10^−5^ K^−1^ (300 K) and in meridianiite [MgSO_4_·11H_2_O (Fortes *et al.*, 2008 ▸] it is 7.2 × 10^−5^ K^−1^ (250 K).

As a major result of the present investigation, we observed no evidence suggesting a structural phase transition in any of the four investigated compounds within the studied temperature range, *i.e.* all four title compounds keep the monoclinic *C*2/*c* symmetry that the kieserite structure type features under ambient conditions. Note that a magnetic order–disorder transition was reported for FeSO_4_·H_2_O at 29.6 K using Mössbauer spectroscopy (Van Alboom *et al.*, 2009 ▸), which is, however, not expected to be detectable in X-ray diffraction studies. Bearing in mind that high-pressure phase transitions in the investigated compounds occur at critical pressures *P*_c_ ≥ 2.40 GPa (Meusburger *et al.*, 2019 ▸, 2020 ▸; Ende *et al.*, 2020 ▸; Wildner *et al.*, 2021 ▸), corresponding to cell volume ratios *V*/*V*_0_ ≤ 0.96, whereas the intrinsic ‘thermal pressure’ (*e.g.* Anderson, 1995 ▸) induced on cooling from RT to 15 K merely results in a volume decrease to roughly *V*/*V*_0_ ≃ 0.993 (Fig. S1 of the supporting information), it becomes clear that phase transitions corresponding to those at high pressures were not to be expected. This also means that kieserite-type compounds and very probably also their solid solutions will not undergo structural transitions at or close to the surface of sulfate-bearing celestial bodies. The relationships of changes in cell and crystal chemical parameters on cooling versus pressure increase are further discussed in detail in the respective sections below.

### 3.3. Unit-cell thermal expansion versus compressibility

Compared with the temperature-dependent changes in lattice dimensions discussed above, the high-pressure behavior of the title compounds, investigated recently by Meusburger *et al.* (2019 ▸, 2020 ▸), Ende *et al.* (2020 ▸) and Wildner *et al.* (2021 ▸) is characterized by a continuous second-order ferroelastic phase transition from the present monoclinic α-phase (*C*2/*c*) to a triclinic β-phase (space group *P* 1) at pressures of 2.72, 6.15, 2.40 and 2.47 GPa for *M* ^2+^ = Mg, Fe, Co and Ni, respectively. Nevertheless, since the cells of the β-polymorphs are based on the respective reduced cells of the *C*2/*c* α-phase, a direct comparison of the lattice dimensions even across the phase transition is still feasible. However, such a comparison [see Tables S1, 1 ▸ and Figs. 2 ▸, S1 in the present work, and Table 2 in the work by Wildner *et al.* (2021 ▸)] shows that the ideally expected inverse relationship between compression and thermal expansion (*e.g.* Hazen & Finger, 1982*a* ▸) is approximately fulfilled for selected aspects only (Fig. S1).

On the one hand, the Fe compound revealing the highest volume thermal expansion α_*V*_ (Table 1 ▸) shows, as expected, the highest compressibility and thus the smallest bulk modulus of *K*_0_ ≃ 45 GPa (Meusburger *et al.*, 2019 ▸), albeit close to *K*_0_ of the Mg and Co phases. The Ni compound with the clearly highest bulk modulus *K*_0_ ≃ 60 GPa (Ende *et al.*, 2020 ▸) also exhibits the smallest thermal changes of the axial lengths (〈α_|*a*,*b*,*c*|_〉 ≃ 2.2 × 10^−5^ K^−1^) but a volume thermal expansion comparable to the Mg and Co phases (Table 1 ▸). Among the individual cell axes, the softest *c* axis upon compression (〈*M_c_* 〉 ≃ 125 GPa) most closely follows the expectation of equal slopes in *V*/*V*_0_ versus *l*/*l*_0_ plots for pressure and temperature (〈α*_c_* 〉 ≃ 3.3 × 10^−5^ K^−1^) in Fig. S1. On the other hand, in contrast to the expected inverse relationship, the clearly stiffest *a* axes upon compression (〈*M_a_* 〉 ≃ 395 GPa) exhibit the highest thermal expansion (〈α*_a_* 〉 ≃ 4.4·10^−5^ K^−1^) and, finally, the NTE behavior (〈α*_b_* 〉 ≃ −2.4 × 10^−5^ K^−1^) of the rather ‘soft’ *b* axes (〈*M_b_* 〉 ≃ 145 GPa) is in harsh contradiction to all respective expectations.

The relationship referred to (Hazen & Finger, 1982*a* ▸) is primarily expected for high-temperature structural studies, but the trends in Fig. 2 ▸ can evidently be extrapolated also to moderately higher temperatures, presumably up to the decomposition temperatures [≥300°C under dry conditions (Chipera *et al.*, 2006 ▸)]. Besides, the relationship is expected to fail for structures comprising polyhedra with grossly different ratios of expansivity versus compressibility (*e.g.* Sharp *et al.*, 1987 ▸). While this is seemingly not the case in kieserite-type compounds with tetrahedral S—O bond lengths hardly responding to changes either in temperature (see below) or pressure (Wildner *et al.*, 2021 ▸), the O—H⋯O hydrogen bonds in these compounds run within structural voids which in the topologically related structure of titanite, CaTiO(SiO_4_), are occupied by the Ca atoms (Hawthorne *et al.*, 1987 ▸). Hence, the hydrogen bonds in *M*SO_4_·H_2_O might play a major role for the clear deviation from respective expectations.

### 3.4. Polyhedral thermal expansion and crystal chemical changes

**Figure 4:**
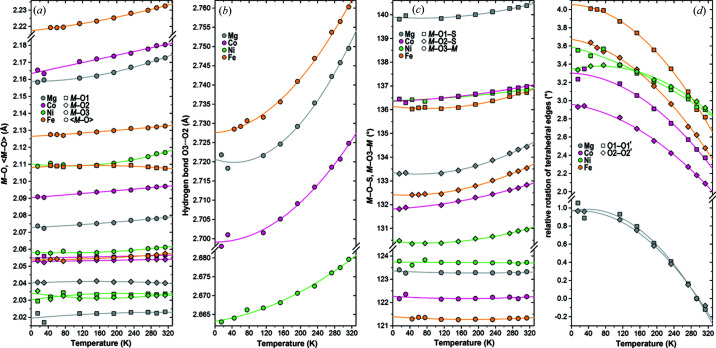
Selected stereochemical parameters of kieserite-type compounds *M* ^2+^SO_4_·H_2_O (*M* ^2+^ = Mg, Fe, Co, Ni) in the temperature range 15–313 K with second-order regression lines. (*a*) Individual and mean *M*—O bond lengths, (*b*) O3—H⋯O2 hydrogen bond lengths, (*c*) *M*—O—S and *M*–O3–*M* angles at polyhedra-linking oxygen atoms, and (*d*) rotations of tetrahedral O1—O1′ and O2—O2′ edges relative to those in the Mg compound at 293 K. For errors see the underlying structural data in Tables S1, S2 and S3.

The octahedral volumes and mean *M*–O bond lengths decrease on cooling by reducing the two longest *M*–O3 bonds to the H_2_O molecules [Fig. 4 ▸(*a*) and Table S3]. For the Fe compound, the bonds to O1 and O2 also seem to show a faint trend to lengthen and shorten, respectively, with reduced temperature; thus, for the FeO_6_ octahedron the aberrant tendency towards a [2+2+2]-coordination (*cf*. Talla & Wildner, 2019 ▸; Wildner *et al.*, 2021 ▸) still increases, compared with the clear and stable [4+2]-elongation of the other representatives. On average, the volumes of the *M*O_6_ octahedra shrink with an α_*V*__oct_ of roughly 2.9 × 10^−5^ K^−1^; in the Mg compound, for example, *V*_oct_ decreases by 0.7% from 11.90 to 11.82 Å^3^ from RT to 15 K, a rate comparable in magnitude to the MgO_6_ octahedra in other magnesium sulfates, as listed by Fortes *et al.* (2008 ▸) – but excluding data from ultra-low *T* < 5 K. Along with the volume reduction, the octahedral bond length and angle distortions also decrease slightly with decreasing temperature for all four compounds.

S—O bond lengths and the tetrahedral volume show an artificial increase on cooling owing to changes in thermal motion (Table S3), a phenomenon typically found for uncorrected bonds in small coordination polyhedra [*e.g.* data for sulfate groups listed by Fortes *et al.* (2008 ▸)]. In the present kieserite-type compounds, a ‘simple rigid bond’ correction according to Downs *et al.* (1992 ▸) reveals almost constant 〈S—O〉 bond lengths over the full temperature range. Anyway, the observed difference of around 0.004 Å in the 〈S—O〉 distances in the Mg compound (shorter S—O bonds) compared with the TM representatives (longer S—O bonds) is maintained over the investigated temperature range. In the same range the O3—H⋯O2 hydrogen bond lengths reduce by roughly 0.03 Å, in case of the Ni phase by half that value [Fig. 4 ▸(*b*) and Table S3]. Somewhat surprisingly, the two O3—H⋯O1 contacts (around 3.3 Å under ambient conditions), which shorten under pressure (one of them forming a new hydrogen bond in the high-pressure β-*M*SO_4_·H_2_O polymorphs), significantly lengthen when the temperature is reduced. Since the O3—H⋯O1 contacts run nearly parallel to the *b* axis, this unexpected finding can be attributed to a side-effect of the axial NTE behavior. Polyhedra-linking angles [Fig. 4 ▸(*c*) and Table S3] also show a parallel response to temperature in all four compounds, despite the fact that both *M*–O–S angles (but especially *M*–O1–S) are substantially larger in MgSO_4_·H_2_O than in the TM phases, leading to, among other things, a volume mismatch of octahedral versus cell volume, especially eye-catching when comparing the Mg and Co compounds [as discussed in detail by Bechtold & Wildner (2016 ▸)]. While the octahedral chain angle *M*–O3–*M* remains almost constant on cooling to 15 K, the *M*–O–S angles become smaller by 0.4–1.1°. The resulting rotation of the SO_4_ tetrahedron around its twofold symmetry axis [Figs. 4 ▸(*d*) and 5 ▸] has the same direction as that found for increasing pressure (Wildner *et al.*, 2021 ▸) or increasing *x*_TM_ in (Mg,TM)SO_4_·H_2_O solid solutions (Bechtold & Wildner, 2016 ▸; Talla & Wildner, 2019 ▸; Talla *et al.*, 2020 ▸). Within the studied temperature range, it amounts to roughly 1.1° for both O1—O1′ and O2—O2′ tetrahedral edges, but again clearly less in the Ni phase. Substantial edge tiltings or rotations of the four other tetrahedral (O1—O2) edges, identified as the most probable driving force of the high-pressure phase transition to triclinic symmetry (Wildner *et al.*, 2021 ▸), are either forbidden or at least strongly hampered by the monoclinic symmetry prevailing also at very low temperatures [*e.g.* relative rotations of O1—O2 edges are <0.1° and therefore not shown in Fig. 4 ▸(*d*)].

The striking anisotropy of the thermal expansion tensor in the *a* *c* plane [Figs. 3 ▸(*a*) and 3(*b*)] can be correlated to the tetrahedral rotations described above. The sense of rotation on heating [indicated in Fig. 3 ▸(*b*) by black arrows] leads to, apart from comparable expansions of the *a* and *c* axes (Table 1 ▸), an opposite shift of neighboring octahedral–tetrahedral chains along the *c* axes [red arrows in Fig. 3 ▸(*b*)], resulting in a widening of the cell angle β (Fig. 2 ▸) and maximum expansion roughly along the long cell diagonal, while minimum expansion occurs near the [101] direction.

**Figure 5:**
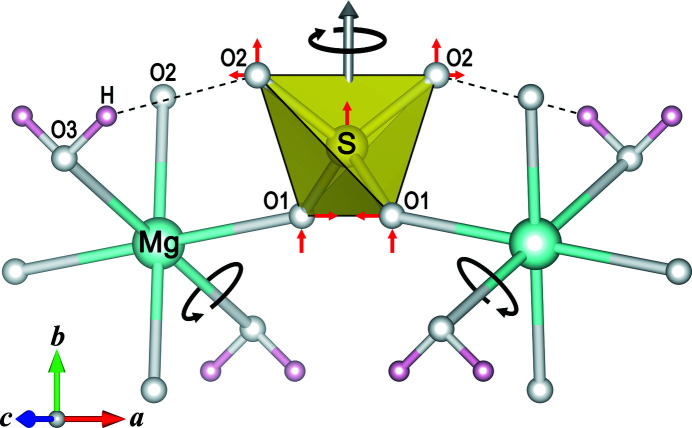
Fragment of the crystal structure of kieserite (MgSO_4_·H_2_O) at 313 K, projected down the approximate c* direction, *i.e.* along [203]. On cooling, the black arrows show the direction of polyhedral rotations, red arrows indicate the components of the resulting positional shifts of the tetrahedral atoms in the plane of projection (see text).

Although the anisotropy of thermal expansion in the *a* *c* plane can be readily explained, an attempt to elucidate reasons for the peculiar NTE behavior of the crystallographic *b* axis [Fig. 3 ▸(*a*), Table 1 ▸] needs to consider the mutual interactions of the various temperature-dependent structural changes observed in the present study. It appears that only a complex, cooperative effect involving polyhedral rotations, changes of the interpolyhedral *M*–O–S angles and shortening of the O3—H⋯O2 hydrogen bond length, can provoke this clear trend in contrast to the decrease in cell volume and other lattice directions on cooling. Fig. 5 ▸ illustrates the main contributing factors: firstly, the sense of major polyhedral rotations on cooling is shown, *i.e.* on the one hand the rotation of the SO_4_ tetrahedron around its twofold symmetry axis [as discussed above; Figs. 3 ▸(*b*) and 4 ▸(*d*)], on the other hand a rotation of the *M*O_6_ octahedron (point symmetry 1) approximately around its O3—O3 axis; secondly, it indicates the most relevant components of resulting positional shifts of the tetrahedral atoms in the plane of projection. Altogether, the SO_4_ tetrahedron as a whole is shifted up along the *b* axis, thus involving an elongation of this axis on cooling, while at the same time the *a* axis, including the roughly parallel hydrogen bond, and to a lesser extent the *c* axis, are shortened. The comparatively weak NTE of the *b* axis in NiSO_4_·H_2_O (Table 1 ▸) can then be correlated, at least qualitatively, with the respective smallest temperature-dependent changes of the hydrogen bond lengths [Fig. 4 ▸(*b*)], of both *M*–O–S angles [Fig. 4 ▸(*c*)] and of the tetrahedral rotations [Fig. 4 ▸(*d*)] among the four title compounds.

### 3.5. Polyhedral thermal expansion versus compressibility

In analogy with the present low-temperature results, the decrease of the octahedral volume on pressure increase is also dominated by shortening of the longest *M*–O3 bonds to the water molecules, to a lesser extent also the *M*–O2 distances, while the *M*–O1 bonds are the stiffest and shorten the least [thus contributing to the high axial 〈*M_a_* 〉 values (Wildner *et al.*, 2021 ▸, and references therein)]. When the octahedral volume thermal expansion values α_*V*,__oct_ (in the range 2.8–3.1 × 10^−5^ K^−1^) are compared with the respective octahedral volume compression values β*_V,_*_oct_ (1.1–1.4 × 10^−6^ bar^−1^), we find a ratio α_*V*,oct_/β_*V*,oct_ of ∼25 bar K^−1^, *i.e.* for the *M*O_4_(H_2_O)_2_ polyhedra a pressure change of approximately 25 bar offsets a temperature change of 1 K. Hence, judging from the influence of temperature and pressure on the octahedral units in kieserite-type compounds, cooling from RT to 15 K corresponds to a ‘thermal pressure effect’ of ∼0.7 GPa, thus far below the magnitude of critical pressures mentioned earlier, and a further corroboration of the absence of structural phase transitions on cooling.

Across high-temperature and high-pressure studies on structure types of relevant rock-forming minerals, the ratio of α_*V*,oct_/β_*V*,oct_ for most *M* ^2+^O_6_ octahedra is about three times higher than in kieserite, scattering between 65 and 90 bar K^−1^ (*e.g.* Hazen & Prewitt, 1977 ▸; Hazen & Finger, 1982*a* ▸,*b* ▸; Hazen *et al.*, 2000 ▸). On the one hand, this difference can be attributed to the fact that α_poly_ increases with temperature: for high-temperature studies from RT up to 1000°C, Hazen & Prewitt (1977 ▸) found 〈α*_V,_*_oct_〉 ≃ 4.2 × 10^−5^ K^−1^ (from axial α_oct_ ≃ 1.4 × 10^−5^ K^−1^) for MgO_6_ octahedra (and similar values for TM^2+^O_6_ units). On the other hand, the compressibility β_*V*,oct_ of the MgO_6_ octahedron in kieserite (Meusburger *et al.*, 2020 ▸; Wildner *et al.*, 2021 ▸) is approximately twice as large compared with the values usually found for MgO_6_ units in oxides and silicates [where the polyhedral modulus *K*_oct_ ≃ 150 GPa (*e.g.* Hazen *et al.*, 2000 ▸)], a discrepancy which can be reasonably explained by the presence of H_2_O ligands and polyhedral connectivity through shared corners in the kieserite structure type.

## 4. Conclusions

The results for the *in situ* investigation of the crystal behavior of kieserite and three sulfate analog phases revealed that the structural topology remained unchanged within the experimental temperature range, *i.e.* down to 15 K. Neither the bond-length evolution nor the overall symmetry of the lattice is subject to a discontinuity that can be assigned to a phase transformation similar to that observed at high pressures for any of the four investigated *M* ^2+^SO_4_·H_2_O (*M* ^2+^ = Mg, Fe, Co, Ni) end-member phases. The monoclinic *C*2/*c* lattice of this so-called α-form shows a rather uniform volume expansivity with values for α_*V*_ between 3.3 and 4.7 × 10^−5^ K^−1^, approximately following the inverse relationship to the molar volume and the size of the *M* ^2+^ cation, respectively. On the other hand, the thermally induced lattice expansion is subject to a rather pronounced anisotropy, with an NTE along the [010] direction, and anisotropic positive eigenvalues for the strain tensor within the *ac* plane. The NTE effect can be related to cooperative polyhedral rotations associated with the chain motifs of tetrahedral SO_4_ groups and octahedrally coordinated *M* ^2+^ cations, which has also been observed for other sulfates exhibiting equivalent topological octahedral chain units.

As a major result of the present investigations, there is no evidence for a structural transition for any of the four investigated end-member compounds. This suggests that, for the kieserite-type solid solution series known to exist, there is no evidence for the occurrence of a low-temperature polymorphic transformation or dissociation, and phase stabilities are not affected even by the effect of cation substitution on temperature variations at ≤1 bar ‘atmospheric’ pressure. In the context of evaluating structural aspects of phase-stability issues with respect to potentially changing hydration states, the experiments clearly show that there is no evidence for any changes with respect to the amount of water per formula unit.

The knowledge of phase relations and structural details at astrophysically relevant temperature conditions is an important fundament to derive, model and analyze other physical properties, *e.g.* by using density functional theory calculations. Based on the low-temperature structural behavior presented, *ab initio* calculations allow us to determine key thermodynamic properties of the phase of interest, such as heat of formation, enthalpy, entropy and the heat capacity (*e.g.* Deffrennes *et al.*, 2019 ▸; Dinsdale *et al.*, 2019 ▸; Bao *et al.*, 2021 ▸). In turn, such as in the case of kieserite-type sulfate monohydrates, knowledge of these properties for end members allows us to extrapolate their values even for intermediate compositions, given the linear Vegard-type behavior along the solid solutions between kieserite and all isotypic TM end members investigated here (Bechtold & Wildner, 2016 ▸; Talla & Wildner, 2019 ▸; Talla *et al.*, 2020 ▸). Knowledge of these parameters will contribute to future modeling of thermodynamic equilibria and stable surface mineral assemblages on various objects in our solar system even at very low temperatures. Last but not least, the absence of low-temperature structural phase transitions or dehydration effects on the one hand, and the smooth variation of stereochemical details with temperature on the other, also forms an important fundament to evaluate kieserite-related signals in remote-sensing spectroscopic data from orbiters at celestial bodies with lower surface temperatures than prevailing on Mars, *e.g.* on the surface on the icy Galilean moons.

## Supplementary Material

Crystal structure: contains datablock(s) publication_text, MgSO4H2O_He_15K, MgSO4H2O_He_30K, MgSO4H2O_N2_113K, MgSO4H2O_N2_153K, MgSO4H2O_N2_193K, MgSO4H2O_N2_233K, MgSO4H2O_N2_273K, MgSO4H2O_N2_313K, FeSO4H2O_He_45K, FeSO4H2O_He_60K, FeSO4H2O_He_75K, FeSO4H2O_N2_113K, FeSO4H2O_N2_153K, FeSO4H2O_N2_193K, FeSO4H2O_N2_233K, FeSO4H2O_N2_273K, FeSO4H2O_N2_313K, CoSO4H2O_He_15K, CoSO4H2O_He_30K, CoSO4H2O_N2_113K, CoSO4H2O_N2_153K, CoSO4H2O_N2_193K, CoSO4H2O_N2_233K, CoSO4H2O_N2_273K, CoSO4H2O_N2_313K, NiSO4H2O_He_15K, NiSO4H2O_He_45K, NiSO4H2O_He_75K, NiSO4H2O_N2_113K, NiSO4H2O_N2_153K, NiSO4H2O_N2_193K, NiSO4H2O_N2_233K, NiSO4H2O_N2_273K, NiSO4H2O_N2_313K. DOI: 10.1107/S2052252521012720/lt5044sup1.cif

Supporting figures and table. DOI: 10.1107/S2052252521012720/lt5044sup2.pdf

CCDC references: 2067136, 2067139, 2067140, 2125292, 2125293, 2125294, 2125295, 2125296, 2125297, 2125298, 2125299, 2125300, 2125301, 2125302, 2125303, 2125304, 2125305, 2125306, 2125307, 2125308, 2125309, 2125310, 2125311, 2125312, 2125313, 2125314, 2125315, 2125316, 2125317, 2125318, 2125319, 2125320, 2125321, 2125322

## Figures and Tables

**Table 1:** Results of Fei-type thermal EoS and strain tensor calculations for kieserite-type compounds *M* ^2+^SO_4_·H_2_O (*M* ^2+^ = Mg, Fe, Co, Ni) for *T*_ref_ = 293 K using *EoSFit7c* (Angel *et al.*, 2014 ▸) Values of α are given as α × 10^−5^ K^−1^, α_0_ × 10^5^ K^−1^ and α_1_ × 10^8^ K^−2^. The parameter α_2_ was fixed at α_2_ = 0. Strain tensor components *e_ij_* (based on Cartesian axes *X* // a*, *Y* // *b*, *Z* // *c*) are given for 113 K.

	Mg	Fe	Co	Ni
α_*V*,293_	3.41 (65)	4.66 (49)	3.34 (28)	3.55 (37)
*V*_0_	355.75 (5)	365.65 (6)	354.77 (3)	342.98 (3)
α_0(*V*)_	0.5 (7)	0.3 (5)	1.4 (3)	−1.7 (4)
α_1(*V*)_	10 (3)	15 (3)	6.7 (15)	18 (2)
				
α_*a*,293_	5.89 (24)	4.49 (18)	4.28 (26)	2.76 (54)
*a*_0_	6.9146 (4)	7.0863 (3)	6.9699 (3)	6.8289 (6)
α_0(*a*)_	–0.4 (5)	1.58 (18)	1.2 (3)	0.7 (5)
α_1(*a*)_	21.4 (12)	9.9 (9)	10.4 (13)	7(3)
				
α_*b*,293_	–4.36 (30)	–1.71 (19)	–2.68 (16)	–0.79 (49)
*b*_0_	7.6285 (6)	7.5545 (3)	7.5947 (3)	7.6048 (7)
α_0(*b*)_	0.6 (3)	–0.98 (19)	–0.34 (16)	–1.4 (5)
α_1(*b*)_	–17.0 (15)	–2.5 (10)	–8.0 (8)	2(3)
				
α_*c*,293_	4.05 (21)	3.31 (16)	2.95 (24)	3.04 (40)
*c*_0_	7.6429 (4)	7.7802 (3)	7.6321 (4)	7.4625 (5)
α_0(*c*)_	–0.3 (2)	0.76 (16)	1.8 (2)	–1.3 (4)
α_1(*c*)_	14.8 (10)	8.7 (8)	4.0 (12)	15 (2)
				
*e*_11_	–0.00468 (6)	–0.00416 (6)	–0.00390 (7)	–0.00216 (14)
*e*_22_	0.00522 (6)	0.00279 (6)	0.00339 (7)	0.00172 (13)
*e*_33_	–0.00491 (6)	–0.00452 (7)	–0.00446 (8)	–0.00342 (14)
*e*_13_	0.00300 (3)	0.00266 (3)	0.00214 (3)	0.00155 (6)
