# Supplementary material for: Crystallography relevant to Mars and Galilean icy moons: crystal behavior of kieserite-type monohydrate sulfates at extraterrestrial conditions down to 15 K
Source: IUCrJ. 2022 Jan 11;9(Pt 2):194–203. doi: 10.1107/S2052252521012720 (PMC8895014; doi:10.1107/S2052252521012720)
Supplement: Supplementary file 2 [file m-09-00194-sup2.pdf]

IUCrJ

Volume 9 (2022)

Supporting information for article:

Crystallography relevant to Mars and Galilean icy moons: Crystal behavior of kieserite-type monohydrate sulfates at extraterrestrial conditions down to 15 Kelvin

Manfred Wildner, Boris A. Zakharov, Nikita E. Bogdanov, Dominik Talla, Elena V. Boldyreva and Ronald Miletich

Figure S1. Relative unit cell dimensions of kieserite-type compounds $M^{2+}SO_4{\cdot}H_2O$ (M^{2+} = Mg, Fe, Co, Ni) as a function of temperature (upper right plot) and pressure (lower left plot, data from Meusburger *et al.*, 2019, 2020, Ende *et al.*, 2020, and Wildner *et al.*, 2021). Note that the plot referring to temperature is scaled to 500%. Linear regression lines are shown to guide the eye. For errors see the underlying structural data in Tables S1 and S2 and in the references cited above.

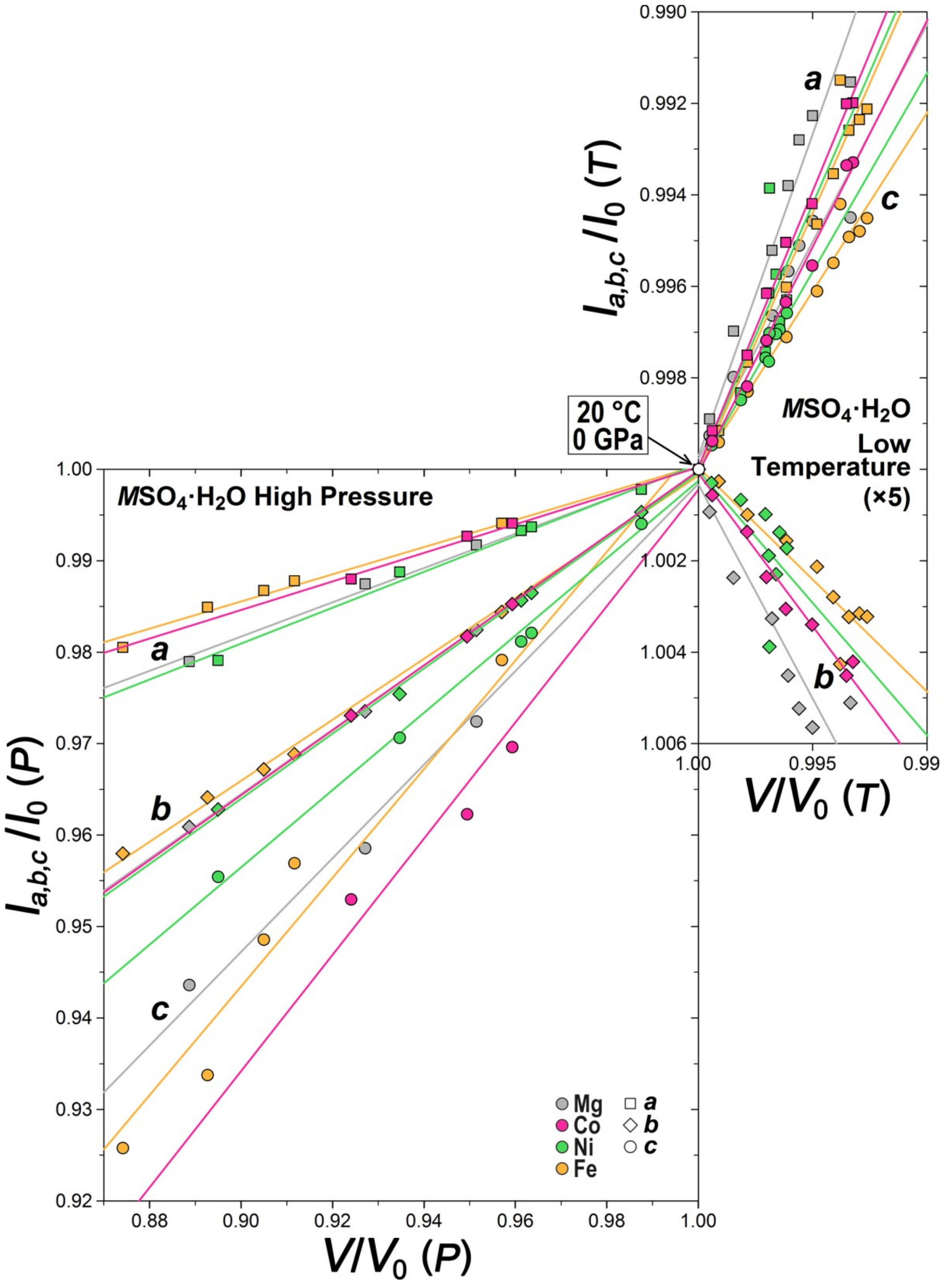

Table S1. Selected crystal data and details of the data collections and structure refinements for kieserite-type compounds $M^{2+}SO_4{\cdot}H_2O$ (M^{2+} = Mg, Fe, Co, Ni) between 15 and 313 K.

**$MgSO_4{\cdot}H_2O$** T (K)	313	273	233	193	153	113	30	15
a (Å) [a]	6.9227(3)	6.9070(3)	6.8937(3)	6.8815(3)	6.8717(3)	6.8648(3)	6.8560(9)	6.8611(6)
b (Å) [a]	7.6212(3)	7.6356(3)	7.6466(3)	7.6534(3)	7.6629(3)	7.6684(3)	7.6675(8)	7.6716(6)
c (Å) [a]	7.6488(3)	7.6373(3)	7.6275(3)	7.6172(3)	7.6098(3)	7.6055(3)	7.6008(10)	7.6014(7)
β (°) [a]	118.11(1)	118.02(1)	117.94(1)	117.88(1)	117.83(1)	117.79(1)	117.82(2)	117.79(1)
V (Å^3)	355.94(2)	355.57(2)	355.20(2)	354.60(2)	354.35(3)	354.18(3)	353.38(7)	353.97(5)
μ (mm^{-1})	0.97	0.97	0.97	0.97	0.97	0.97	0.97	0.97
D_{calc} (gcm^{-3})	2.582	2.585	2.588	2.592	2.594	2.595	2.601	2.597
Total number of reflections	10441	10423	10356	10428	10369	10307	1112	1294
Intensity data for unit cell	6121	6673	6681	6649	6917	7061	762	886
Unique hkl's	1103	1101	1100	1098	1098	1097	410	422
R_i (%)	2.09	1.87	1.94	1.96	1.87	1.84	7.12	2.30
$F_o > 4\sigma(F_o)$	1049	1058	1050	1047	1049	1054	352	381
wR2 [for all F_o^2] (%)	4.84	4.57	4.45	4.48	4.38	4.26	12.67	5.77
weighting parameter a, b[b]	0.020, 0.22	0.020, 0.17	0.019, 0.19	0.020, 0.18	0.019, 0.18	0.018, 0.21	0.078, 0.00	0.026, 0.47
R1 [for $F_o > 4\sigma(F_o)$] (%)	1.72	1.57	1.51	1.56	1.51	1.48	5.05	2.30
R1 [for all F_o] (%)	1.85	1.66	1.63	1.67	1.60	1.56	5.88	2.66
Goodness of fit	1.180	1.211	1.200	1.202	1.232	1.207	1.051	1.112
Extinction coefficient	0.004(2)	0.005(2)	0.005(2)	0.004(2)	0.005(2)	0.006(2)	0.132(18)	0.077(5)
$\Delta\rho_{max, min}$ (e^- Å^{-3})	0.47, -0.37	0.52, -0.37	0.52, -0.41	0.49, -0.37	0.48, -0.41	0.46, -0.40	0.82, -0.96	0.41, -0.50

[a] lattice parameters are corrected to comply with respective data from Nonius Kappa CCD room temperature measurement (see Experimental section).

[b] $w = 1 / [\sigma^2(F_o^2) + (a \times P)^2 + b \times P]$; $P = \{[\max \text{ of } (0 \text{ or } F_o^2)] + 2F_c^2\} / 3$.

**$FeSO_4{\cdot}H_2O$** T (K)	313	273	233	193	153	113	75	60	45	15*
a (Å) [a]	7.0923(5)	7.0802(3)	7.0696(3)	7.0580(3)	7.0482(3)	7.0404(3)	7.0337(9)	7.0320(8)	7.0304(8)	7.0259(11)
b (Å) [a]	7.5523(5)	7.5565(4)	7.5620(3)	7.5663(3)	7.5706(4)	7.5756(4)	7.5789(7)	7.5783(6)	7.5789(6)	7.5867(11)
c (Å) [a]	7.7848(5)	7.7755(4)	7.7669(3)	7.7576(3)	7.7498(4)	7.7450(4)	7.7406(9)	7.7396(8)	7.7374(8)	7.7350(11)
β (°) [a]	118.65(1)	118.58(1)	118.52(1)	118.45(1)	118.40(1)	118.37(1)	118.33(2)	118.33(1)	118.31(1)	118.20(2)
V (Å^3)	365.93(7)	365.31(5)	364.85(5)	364.23(5)	363.74(5)	363.48(5)	363.23(7)	363.06(6)	362.94(6)	363.37(9)
μ (mm^{-1})	4.58	4.58	4.59	4.60	4.60	4.61	4.61	4.61	4.61	
D_{calc} (gcm^{-3})	3.084	3.090	3.094	3.099	3.103	3.105	3.107	3.109	3.110	
Total number of reflections	10941	10880	10878	10851	10827	10783	1255	1248	1250	
Intensity data for unit cell	7887	8198	8419	8498	8632	8607	995	998	997	297
Unique hkl's	1139	1137	1133	1131	1130	1128	445	442	442	
R_i (%)	3.64	3.20	3.06	2.60	2.33	2.30	1.90	2.06	1.99	
$F_o > 4\sigma(F_o)$	1115	1117	1117	1112	1118	1118	425	423	425	
wR2 [for all F_o^2] (%)	3.79	3.60	3.53	3.38	3.32	3.22	5.16	4.36	4.25	
weighting parameter a, b[b]	0.018, 0.15	0.018, 0.17	0.017, 0.17	0.015, 0.20	0.014, 0.24	0.013, 0.24	0.029, 0.14	0.020, 0.31	0.021, 0.34	
R1 [for $F_o > 4\sigma(F_o)$] (%)	1.46	1.37	1.32	1.28	1.28	1.27	1.87	1.77	1.72	
R1 [for all F_o] (%)	1.50	1.40	1.34	1.31	1.30	1.29	2.00	1.88	1.77	
Goodness of fit	1.128	1.101	1.146	1.168	1.182	1.193	1.120	1.094	1.121	
Extinction coefficient	0.209(4)	0.231(4)	0.079(2)	0.033(1)	0.022(1)	0.042(1)	0.035(2)	0.033(2)	0.035(2)	
$\Delta\rho_{max, min}$ (e$^-$ Å^{-3})	0.51, -0.71	0.51, -0.83	0.55, -0.54	0.49, -0.52	0.55, -0.50	0.68, -0.44	0.62, -0.55	0.51, -0.44	0.54, -0.47	

* only lattice parameters, no structure refinement

[a] lattice parameters are corrected to comply with respective data from Nonius Kappa CCD room temperature measurement (see Experimental section).

[b] $w = 1 / [\sigma^2(F_o^2) + (a{\times}P)^2 + b{\times}P]$; $P = \{[\max of (0 or F_o^2)] + 2F_c^2\} / 3$.

$CoSO_4 \cdot H_2O$ T (K)	313	273	233	193	153	113	30	15
a (Å) [a]	6.9765(4)	6.9641(4)	6.9526(4)	6.9432(4)	6.9354(4)	6.9295(4)	6.9142(10)	6.9143(10)
b (Å) [a]	7.5904(4)	7.5990(4)	7.6051(4)	7.6126(4)	7.6179(4)	7.6205(5)	7.6267(8)	7.6290(8)
c (Å) [a]	7.6373(4)	7.6275(4)	7.6184(4)	7.6107(4)	7.6043(4)	7.5982(5)	7.5810(10)	7.5815(10)
β (°) [a]	118.62(1)	118.55(1)	118.50(1)	118.44(1)	118.40(1)	118.38(1)	118.18(2)	118.19(2)
V (Å^3)	355.03(5)	354.56(5)	354.02(5)	353.72(5)	353.42(5)	353.01(6)	352.38(9)	352.48(9)
μ (mm^{-1})	5.30	5.31	5.32	5.32	5.33	5.33	5.34	5.34
D_{calc} (gcm^{-3})	3.237	3.241	3.246	3.249	3.251	3.255	3.261	3.260
Total number of reflections	11992	11964	11947	11894	11922	10933	1297	1289
Intensity data for unit cell	9143	9270	9349	9419	9452	8741	777	769
Unique hkl's	1105	1103	1101	1098	1098	1095	432	428
R_i (%)	1.97	1.94	1.97	1.98	1.97	1.85	3.36	4.88
$F_o > 4\sigma(F_o)$	1094	1095	1095	1089	1089	1086	386	383
wR2 [for all F_o^2] (%)	3.30	3.19	3.19	2.99	2.95	2.84	6.17	6.50
weighting parameter a, b[b]	0.014, 0.20	0.013, 0.26	0.013, 0.28	0.012, 0.28	0.011, 0.32	0.010, 0.32	0.022, 0.85	0.021, 0.36
R1 [for $F_o > 4\sigma(F_o)$] (%)	1.22	1.21	1.22	1.17	1.18	1.16	2.85	2.69
R1 [for all F_o] (%)	1.24	1.24	1.23	1.19	1.20	1.18	3.37	3.31
Goodness of fit	1.207	1.160	1.161	1.135	1.139	1.147	1.113	1.146
Extinction coefficient	0.024(1)	0.023(1)	0.022(1)	0.021(1)	0.018(1)	0.022(1)	0.020(2)	0.020(2)
$\Delta\rho_{max, min}$ (e$^-$ Å^{-3})	0.44, -0.41	0.47, -0.42	0.47, -0.41	0.48, -0.46	0.52, -0.48	0.50, -0.43	0.80, -0.80	0.83, -0.71

[a] lattice parameters are corrected to comply with respective data from Nonius Kappa CCD room temperature measurement (see Experimental section).

[b] $w = 1 / [\sigma^2(F_o^2) + (a\times P)^2 + b\times P]$; $P = \{[\max \text{ of } (0 \text{ or } F_o^2)] + 2F_c^2\} / 3$.

**$NiSO_4{\cdot}H_2O$** *T* (K)	313	273	233	193	153	113	75	45	15
a (Å) [a]	6.8332(4)	6.8252(4)	6.8176(5)	6.8115(7)	6.8069(8)	6.8037(9)	6.8027(9)	6.7999(8)	6.7870(11)
b (Å) [a]	7.6026(5)	7.6070(4)	7.6098(5)	7.6122(8)	7.6152(9)	7.6178(10)	7.6191(7)	7.6222(6)	7.6342(9)
c (Å) [a]	7.4669(4)	7.4587(4)	7.4513(5)	7.4444(8)	7.4398(9)	7.4371(10)	7.4450(10)	7.4405(9)	7.4404(12)
β (°) [a]	117.78(1)	117.72(1)	117.68(1)	117.63(1)	117.60(1)	117.58(1)	117.61(2)	117.58(2)	117.51(2)
V (Å^3)	343.21(6)	342.79(5)	342.35(7)	341.98(10)	341.77(12)	341.66(13)	341.93(7)	341.82(6)	341.92(9)
μ (mm^{-1})	6.14	6.14	6.15	6.16	6.16	6.16	6.16	6.16	6.14
D_{calc} (gcm^{-3})	3.344	3.348	3.352	3.356	3.358	3.359	3.356	3.358	3.345
Total number of reflections	11484	11574	11529	11491	11529	11115	1293	1304	1207
Intensity data for unit cell	8005	8253	8459	8457	8648	8497	991	1003	903
Unique hkl's	1068	1064	1064	1063	1062	1061	424	422	430
R_i (%)	2.47	2.40	2.35	2.32	2.27	2.35	2.72	2.27	3.63
$F_o > 4\sigma(F_o)$	1034	1031	1033	1036	1034	1034	395	400	398
wR2 [for all F_o^2] (%)	4.19	4.11	4.37	4.20	4.13	4.22	5.21	5.13	6.61
weighting parameters a, b[b]	0.021, 0.33	0.021, 0.34	0.023, 0.40	0.021, 0.47	0.021, 0.48	0.021, 0.53	0.026, 0.00	0.029, 0.09	0.035, 0.00
R1 [for $F_o > 4\sigma(F_o)$] (%)	1.59	1.54	1.63	1.60	1.55	1.61	2.09	1.99	2.60
R1 [for all F_o] (%)	1.66	1.62	1.72	1.67	1.61	1.66	2.27	2.12	2.80
Goodness of fit	1.133	1.127	1.128	1.118	1.110	1.123	1.120	1.106	1.107
Extinction coefficient	0.027(1)	0.025(1)	0.022(1)	0.022(1)	0.020(1)	0.019(1)	0.041(2)	0.043(3)	0.033(3)
$\Delta\rho_{max, min}$ (e^- Å^{-3})	0.71, -0.61	0.67, -0.68	0.85, -0.68	0.80, -0.72	0.78, -0.68	0.82, -0.67	0.44, -0.50	0.55, -0.61	0.88, -0.66

[a] lattice parameters are corrected to comply with respective data from Nonius Kappa CCD room temperature measurement (see Experimental section).

[b] $w = 1 / [\sigma^2(F_o^2) + (a\times P)^2 + b\times P]$; $P = \{[\max \text{ of } (0 \text{ or } F_o^2)] + 2F_c^2\} / 3$.

Table S2. Final atomic parameters for kieserite-type compounds $M^{2+}SO_4 \cdot H_2O$ (M^{2+} = Mg, Fe, Co, Ni) between 15 and 313 K.

$MgSO_4 \cdot H_2O$

**15 K**	*x*	*y*	*z*	U_{iso}*/U_{eq}	U^{11}	U^{22}	U^{33}	U^{12}	U^{13}	U^{23}
Mg	0	0.5	0	0.0038(3)	0.0035(4)	0.0038(5)	0.0049(4)	0.0000(3)	0.0025(3)	0.0005(3)
S	0	0.15743(8)	0.25	0.0030(2)	0.0030(3)	0.0029(4)	0.0037(3)	0	0.0022(2)	0
O1	0.17523(19)	0.04823(17)	0.39724(17)	0.0049(3)	0.0040(6)	0.0042(7)	0.0062(6)	0.0010(5)	0.0022(5)	0.0011(5)
O2	0.09598(19)	0.27005(17)	0.15141(18)	0.0048(3)	0.0042(6)	0.0041(7)	0.0069(6)	0.0000(5)	0.0034(5)	0.0012(5)
O3	0	0.6334(3)	0.25	0.0049(4)	0.0037(9)	0.0040(9)	0.0065(9)	0	0.0020(7)	0
H	0.101(4)	0.700(4)	0.288(4)	0.021(7)*						

**30 K**	*x*	*y*	*z*	U_{iso}*/U_{eq}	U^{11}	U^{22}	U^{33}	U^{12}	U^{13}	U^{23}
Mg	0	0.5	0	0.0057(5)	0.0047(8)	0.0041(9)	0.0093(8)	-0.0002(5)	0.0042(6)	0.0002(5)
S	0	0.15735(13)	0.25	0.0046(5)	0.0037(7)	0.0021(7)	0.0084(7)	0	0.0032(5)	0
O1	0.1756(3)	0.0480(3)	0.3969(3)	0.0073(6)	0.0060(11)	0.0068(13)	0.0096(11)	-0.0001(8)	0.0040(9)	0.0012(8)
O2	0.0959(3)	0.2699(3)	0.1514(3)	0.0070(6)	0.0049(11)	0.0047(12)	0.0123(10)	0.0005(8)	0.0047(9)	0.0010(8)
O3	0	0.6338(4)	0.25	0.0062(8)	0.0039(16)	0.0050(16)	0.0087(16)	0	0.0023(13)	0
H	0.094(7)	0.690(7)	0.284(6)	0.013(11)*						

**113 K**	*x*	*y*	*z*	U_{iso}*/U_{eq}	U^{11}	U^{22}	U^{33}	U^{12}	U^{13}	U^{23}
Mg	0	0.5	0	0.00431(6)	0.00388(11)	0.00445(11)	0.00431(10)	-0.00022(8)	0.00167(8)	0.00014(8)
S	0	0.15710(2)	0.25	0.00337(5)	0.00268(7)	0.00342(7)	0.00377(7)	0	0.00131(5)	0
O1	0.17522(7)	0.04770(6)	0.39686(6)	0.00648(7)	0.00418(14)	0.00732(15)	0.00704(14)	0.00193(11)	0.00186(11)	0.00289(11)
O2	0.09574(7)	0.26975(5)	0.15109(6)	0.00597(7)	0.00588(15)	0.00573(14)	0.00753(14)	0.00023(11)	0.00417(12)	0.00206(11)
O3	0	0.63384(8)	0.25	0.00572(9)	0.0051(2)	0.00623(19)	0.00593(19)	0	0.00259(16)	0
H	0.106(2)	0.6987(18)	0.290(2)	0.019(3)*						

**153 K**	*x*	*y*	*z*	U_{iso}*/U_{eq}	U^{11}	U^{22}	U^{33}	U^{12}	U^{13}	U^{23}
Mg	0	0.5	0	0.00490(6)	0.00441(11)	0.00511(11)	0.00489(11)	-0.00030(8)	0.00194(8)	0.00018(8)
S	0	0.15678(2)	0.25	0.00394(5)	0.00310(7)	0.00392(7)	0.00449(7)	0	0.00152(5)	0
O1	0.17528(7)	0.04724(6)	0.39649(6)	0.00764(7)	0.00485(14)	0.00865(15)	0.00845(15)	0.00234(11)	0.00228(12)	0.00359(12)
O2	0.09519(7)	0.26939(5)	0.15082(6)	0.00695(7)	0.00680(15)	0.00652(14)	0.00900(15)	0.00050(11)	0.00491(12)	0.00257(11)
O3	0	0.63402(8)	0.25	0.00651(9)	0.0059(2)	0.0072(2)	0.0065(2)	0	0.00297(16)	0
H	0.105(2)	0.6999(18)	0.290(2)	0.019(3)*						

**193 K**	*x*	*y*	*z*	U_{iso}*/U_{eq}	U^{11}	U^{22}	U^{33}	U^{12}	U^{13}	U^{23}
Mg	0	0.5	0	0.00566(6)	0.00494(11)	0.00595(11)	0.00571(11)	-0.00034(8)	0.00217(9)	0.00024(8)
S	0	0.15635(2)	0.25	0.00461(5)	0.00351(7)	0.00455(7)	0.00540(7)	0	0.00177(5)	0
O1	0.17541(7)	0.04664(6)	0.39602(7)	0.00910(8)	0.00578(15)	0.01021(16)	0.01016(16)	0.00271(12)	0.00276(13)	0.00436(13)
O2	0.09440(7)	0.26900(6)	0.15044(7)	0.00824(7)	0.00810(16)	0.00758(15)	0.01082(16)	0.00065(12)	0.00592(13)	0.00323(12)
O3	0	0.63431(8)	0.25	0.00757(9)	0.0070(2)	0.0083(2)	0.0075(2)	0	0.00343(17)	0
H	0.104(2)	0.7003(18)	0.291(2)	0.022(3)*						

**233 K**	*x*	*y*	*z*	U_{iso}*/U_{eq}	U^{11}	U^{22}	U^{33}	U^{12}	U^{13}	U^{23}
Mg	0	0.5	0	0.00652(6)	0.00574(11)	0.00680(11)	0.00652(11)	-0.00039(8)	0.00244(9)	0.00034(8)
S	0	0.15586(2)	0.25	0.00530(5)	0.00400(7)	0.00518(7)	0.00623(7)	0	0.00198(5)	0

O1	0.17551(7)	0.04607(6)	0.39547(7)	0.01054(8)	0.00642(15)	0.01185(17)	0.01192(17)	0.00311(13)	0.00312(13)	0.00518(13)
O2	0.09346(7)	0.26857(6)	0.15003(7)	0.00955(8)	0.00931(17)	0.00875(16)	0.01258(17)	0.00087(12)	0.00678(14)	0.00375(12)
O3	0	0.63464(8)	0.25	0.00867(10)	0.0082(2)	0.0095(2)	0.0084(2)	0	0.00396(18)	0
H	0.103(2)	0.7020(18)	0.291(2)	0.024(3)*						

**273 K**	*x*	*y*	*z*	U_{iso}*/U_{eq}	U^{11}	U^{22}	U^{33}	U^{12}	U^{13}	U^{23}
Mg	0	0.5	0	0.00746(6)	0.00655(12)	0.00777(12)	0.00747(11)	-0.00039(8)	0.00280(9)	0.00049(8)
S	0	0.15529(2)	0.25	0.00604(5)	0.00453(7)	0.00592(7)	0.00710(7)	0	0.00226(5)	0
O1	0.17569(7)	0.04536(7)	0.39486(7)	0.01209(8)	0.00720(16)	0.01384(18)	0.01366(17)	0.00361(13)	0.00358(13)	0.00604(14)
O2	0.09235(8)	0.26806(6)	0.14957(7)	0.01101(8)	0.01084(17)	0.01001(16)	0.01447(17)	0.00112(12)	0.00785(14)	0.00436(13)
O3	0	0.63497(9)	0.25	0.00991(10)	0.0095(2)	0.0108(2)	0.0096(2)	0	0.00462(18)	0
H	0.104(2)	0.7024(18)	0.293(2)	0.025(3)*						

**313 K**	*x*	*y*	*z*	U_{iso}*/U_{eq}	U^{11}	U^{22}	U^{33}	U^{12}	U^{13}	U^{23}
Mg	0	0.5	0	0.00852(7)	0.00750(13)	0.00876(13)	0.00858(13)	-0.00057(9)	0.00319(10)	0.00055(9)
S	0	0.15461(3)	0.25	0.00684(5)	0.00519(8)	0.00655(8)	0.00819(8)	0	0.00267(6)	0
O1	0.17585(8)	0.04442(7)	0.39411(8)	0.01380(9)	0.00842(18)	0.0155(2)	0.0157(2)	0.00412(15)	0.00418(15)	0.00690(16)
O2	0.09120(9)	0.26743(7)	0.14908(8)	0.01257(9)	0.0126(2)	0.01123(18)	0.0167(2)	0.00131(14)	0.00915(17)	0.00498(15)
O3	0	0.63534(10)	0.25	0.01125(11)	0.0110(3)	0.0123(3)	0.0110(2)	0	0.0056(2)	0
H	0.103(2)	0.703(2)	0.293(2)	0.028(4)*						

$FeSO_4 \cdot H_2O$

**45 K**	*x*	*y*	*z*	U_{iso}*/U_{eq}	U^{11}	U^{22}	U^{33}	U^{12}	U^{13}	U^{23}
Fe	0	0.5	0	0.00235(16)	0.0025(2)	0.0022(2)	0.0022(2)	0.00025(12)	0.00098(16)	0.00075(11)
S	0	0.15535(7)	0.25	0.00229(17)	0.0019(3)	0.0024(3)	0.0026(3)	0	0.0011(2)	0
O1	0.1683(2)	0.04564(17)	0.40068(17)	0.0046(3)	0.0036(7)	0.0049(6)	0.0045(5)	0.0010(5)	0.0013(5)	0.0008(5)
O2	0.10045(19)	0.27064(15)	0.15967(16)	0.0044(3)	0.0038(7)	0.0042(6)	0.0052(5)	0.0002(5)	0.0023(5)	0.0017(4)
O3	0	0.6435(2)	0.25	0.0045(4)	0.0036(10)	0.0045(9)	0.0056(8)	0	0.0024(7)	0
H	0.101(4)	0.715(3)	0.290(3)	0.020(6)*						

**60 K**	*x*	*y*	*z*	U_{iso}*/U_{eq}	U^{11}	U^{22}	U^{33}	U^{12}	U^{13}	U^{23}
Fe	0	0.5	0	0.00233(16)	0.0024(2)	0.0018(2)	0.0024(2)	0.00016(13)	0.00083(17)	0.00064(12)
S	0	0.15520(8)	0.25	0.00232(18)	0.0020(3)	0.0020(3)	0.0030(3)	0	0.0012(2)	0
O1	0.1684(2)	0.04575(18)	0.40074(19)	0.0050(3)	0.0038(7)	0.0051(6)	0.0055(6)	0.0007(5)	0.0018(5)	0.0012(5)
O2	0.1003(2)	0.27048(16)	0.15946(17)	0.0043(3)	0.0037(7)	0.0041(7)	0.0056(6)	0.0005(5)	0.0028(5)	0.0020(5)
O3	0	0.6434(3)	0.25	0.0043(4)	0.0034(10)	0.0044(9)	0.0046(9)	0	0.0015(8)	0
H	0.102(4)	0.711(3)	0.294(3)	0.012(6)*						

**75 K**	*x*	*y*	*z*	U_{iso}*/U_{eq}	U^{11}	U^{22}	U^{33}	U^{12}	U^{13}	U^{23}
Fe	0	0.5	0	0.00280(18)	0.0025(3)	0.0027(3)	0.0028(2)	0.00005(13)	0.00097(18)	0.00058(12)
S	0	0.15532(8)	0.25	0.0027(2)	0.0021(4)	0.0026(3)	0.0030(3)	0	0.0010(3)	0
O1	0.1686(2)	0.0458(2)	0.40091(19)	0.0052(3)	0.0039(7)	0.0047(7)	0.0060(6)	0.0012(6)	0.0016(5)	0.0018(5)
O2	0.1002(2)	0.27064(17)	0.15934(18)	0.0049(3)	0.0041(7)	0.0047(7)	0.0062(6)	0.0005(5)	0.0027(5)	0.0020(5)
O3	0	0.6434(3)	0.25	0.0046(4)	0.0039(10)	0.0050(10)	0.0046(9)	0	0.0017(8)	0
H	0.098(4)	0.712(3)	0.291(3)	0.011(6)*						

**113 K**	*x*	*y*	*z*	U_{iso}*/U_{eq}	U^{11}	U^{22}	U^{33}	U^{12}	U^{13}	U^{23}
Fe	0	0.5	0	0.00387(4)	0.00364(5)	0.00378(5)	0.00416(5)	0.00003(3)	0.00182(4)	0.00046(3)
S	0	0.15512(2)	0.25	0.00351(4)	0.00283(7)	0.00326(7)	0.00436(7)	0	0.00162(5)	0
O1	0.16853(7)	0.04497(6)	0.40047(7)	0.00700(7)	0.00455(14)	0.00773(16)	0.00792(15)	0.00210(12)	0.00231(12)	0.00347(12)
O2	0.09991(7)	0.27015(6)	0.15910(6)	0.00624(7)	0.00607(15)	0.00564(15)	0.00832(15)	0.00030(11)	0.00450(13)	0.00221(12)
O3	0	0.64381(8)	0.25	0.00616(9)	0.0060(2)	0.0064(2)	0.0064(2)	0	0.00316(17)	0
H	0.109(2)	0.712(2)	0.292(2)	0.021(3)*						

**153 K**	*x*	*y*	*z*	U_{iso}*/U_{eq}	U^{11}	U^{22}	U^{33}	U^{12}	U^{13}	U^{23}
Fe	0	0.5	0	0.00477(4)	0.00440(5)	0.00465(5)	0.00517(5)	0.00000(3)	0.00221(4)	0.00056(3)
S	0	0.15482(2)	0.25	0.00421(4)	0.00323(7)	0.00388(7)	0.00537(7)	0	0.00192(5)	0
O1	0.16858(7)	0.04450(7)	0.40013(7)	0.00847(7)	0.00528(15)	0.00941(17)	0.00988(16)	0.00255(13)	0.00292(13)	0.00441(13)
O2	0.09931(7)	0.26982(6)	0.15882(7)	0.00747(7)	0.00714(15)	0.00668(15)	0.01017(16)	0.00053(12)	0.00539(13)	0.00284(12)
O3	0	0.64402(9)	0.25	0.00722(9)	0.0070(2)	0.0074(2)	0.0076(2)	0	0.00373(18)	0
H	0.107(2)	0.716(2)	0.294(2)	0.025(4)*						

**193 K**	*x*	*y*	*z*	U_{iso}*/U_{eq}	U^{11}	U^{22}	U^{33}	U^{12}	U^{13}	U^{23}
Fe	0	0.5	0	0.00582(4)	0.00531(5)	0.00572(5)	0.00630(5)	-0.00003(3)	0.00266(4)	0.00064(3)
S	0	0.15447(2)	0.25	0.00507(4)	0.00382(7)	0.00462(7)	0.00651(7)	0	0.00225(5)	0
O1	0.16874(7)	0.04405(7)	0.39968(7)	0.01016(8)	0.00629(15)	0.01111(17)	0.01199(17)	0.00299(13)	0.00347(13)	0.00538(14)
O2	0.09854(7)	0.26941(6)	0.15838(7)	0.00889(7)	0.00832(15)	0.00798(15)	0.01216(17)	0.00074(12)	0.00633(13)	0.00344(13)
O3	0	0.64418(9)	0.25	0.00850(9)	0.0084(2)	0.0088(2)	0.0087(2)	0	0.00439(18)	0

H	0.108(2)	0.716(2)	0.294(2)	0.028(4)*						

**233 K**	*x*	*y*	*z*	U_{iso}*/U_{eq}	U^{11}	U^{22}	U^{33}	U^{12}	U^{13}	U^{23}
Fe	0	0.5	0	0.00700(4)	0.00634(5)	0.00685(6)	0.00754(6)	-0.00004(3)	0.00310(4)	0.00074(3)
S	0	0.15406(2)	0.25	0.00601(4)	0.00456(7)	0.00540(7)	0.00768(7)	0	0.00260(5)	0
O1	0.16893(7)	0.04355(7)	0.39918(7)	0.01200(8)	0.00723(15)	0.01321(18)	0.01418(18)	0.00356(14)	0.00400(13)	0.00646(15)
O2	0.09766(7)	0.26893(6)	0.15794(7)	0.01060(8)	0.01009(16)	0.00930(16)	0.01444(18)	0.00102(12)	0.00749(14)	0.00417(13)
O3	0	0.64450(9)	0.25	0.00989(10)	0.0099(2)	0.0102(2)	0.0099(2)	0	0.00501(18)	0
H	0.105(3)	0.718(2)	0.295(2)	0.034(4)*						

**273 K**	*x*	*y*	*z*	U_{iso}*/U_{eq}	U^{11}	U^{22}	U^{33}	U^{12}	U^{13}	U^{23}
Fe	0	0.5	0	0.00810(4)	0.00734(6)	0.00813(6)	0.00863(6)	-0.00008(3)	0.00365(4)	0.00083(3)
S	0	0.15360(2)	0.25	0.00688(5)	0.00523(7)	0.00633(7)	0.00873(7)	0	0.00305(5)	0
O1	0.16920(7)	0.04295(7)	0.39860(8)	0.01391(8)	0.00844(16)	0.01536(19)	0.01643(19)	0.00421(14)	0.00475(14)	0.00751(16)
O2	0.09661(7)	0.26840(6)	0.15737(7)	0.01223(8)	0.01166(16)	0.01093(17)	0.01664(19)	0.00136(13)	0.00882(15)	0.00494(14)
O3	0	0.64461(9)	0.25	0.01132(10)	0.0115(2)	0.0120(2)	0.0110(2)	0	0.00587(19)	0
H	0.106(3)	0.718(2)	0.296(2)	0.037(4)*						

**313 K**	*x*	*y*	*z*	U_{iso}*/U_{eq}	U^{11}	U^{22}	U^{33}	U^{12}	U^{13}	U^{23}
Fe	0	0.5	0	0.00928(5)	0.00841(6)	0.00935(6)	0.00991(6)	-0.00014(3)	0.00425(4)	0.00092(3)
S	0	0.15309(3)	0.25	0.00781(5)	0.00596(7)	0.00716(8)	0.00993(8)	0	0.00352(6)	0
O1	0.16949(8)	0.04222(8)	0.39797(8)	0.01594(9)	0.00969(17)	0.0176(2)	0.0190(2)	0.00462(16)	0.00563(15)	0.00856(17)
O2	0.09559(8)	0.26774(7)	0.15682(8)	0.01397(9)	0.01329(18)	0.01247(18)	0.0190(2)	0.00163(14)	0.01008(16)	0.00557(15)
O3	0	0.64483(10)	0.25	0.01298(11)	0.0133(2)	0.0137(3)	0.0127(2)	0	0.0069(2)	0
H	0.107(3)	0.722(3)	0.297(2)	0.040(4)*						

$CoSO_4·H_2O$

**15 K**	*x*	*y*	*z*	U_{iso}*/U_{eq}	U^{11}	U^{22}	U^{33}	U^{12}	U^{13}	U^{23}
Co	0	0.5	0	0.0041(2)	0.0045(3)	0.0025(4)	0.0049(4)	-0.0002(2)	0.0019(3)	0.0002(2)
S	0	0.15579(15)	0.25	0.0042(3)	0.0046(5)	0.0026(6)	0.0050(5)	0	0.0019(4)	0
O1	0.1722(3)	0.0452(3)	0.4025(3)	0.0051(5)	0.0045(10)	0.0025(11)	0.0079(10)	0.0013(9)	0.0026(9)	0.0003(9)
O2	0.1006(3)	0.2687(3)	0.1558(3)	0.0058(5)	0.0060(10)	0.0034(12)	0.0084(10)	0.0003(9)	0.0036(9)	0.0019(8)
O3	0	0.6373(5)	0.25	0.0068(7)	0.0042(15)	0.0062(18)	0.0088(16)	0	0.0021(14)	0
H	0.100(5)	0.697(5)	0.293(5)	0.008(10)*						

**30 K**	*x*	*y*	*z*	U_{iso}*/U_{eq}	U^{11}	U^{22}	U^{33}	U^{12}	U^{13}	U^{23}
Co	0	0.5	0	0.0039(2)	0.0043(3)	0.0028(4)	0.0042(4)	-0.0003(2)	0.0017(3)	0.0002(2)
S	0	0.15584(15)	0.25	0.0041(3)	0.0050(5)	0.0026(6)	0.0052(5)	0	0.0028(4)	0
O1	0.1720(3)	0.0451(3)	0.4027(3)	0.0056(5)	0.0048(10)	0.0049(12)	0.0071(10)	0.0006(9)	0.0027(9)	0.0009(8)
O2	0.1005(3)	0.2688(3)	0.1559(3)	0.0056(5)	0.0065(10)	0.0039(12)	0.0069(10)	0.0001(9)	0.0037(9)	0.0012(8)
O3	0	0.6368(4)	0.25	0.0056(7)	0.0058(15)	0.0043(17)	0.0074(15)	0	0.0037(14)	0
H	0.104(6)	0.695(7)	0.291(6)	0.029(13)*						

**113 K**	*x*	*y*	*z*	U_{iso}*/U_{eq}	U^{11}	U^{22}	U^{33}	U^{12}	U^{13}	U^{23}
Co	0	0.5	0	0.00327(3)	0.00299(5)	0.00348(5)	0.00344(5)	-0.00003(3)	0.00161(3)	0.00024(3)
S	0	0.15532(2)	0.25	0.00313(4)	0.00255(7)	0.00322(7)	0.00371(7)	0	0.00155(5)	0
O1	0.17207(7)	0.04480(6)	0.40193(7)	0.00652(7)	0.00425(14)	0.00740(16)	0.00727(16)	0.00203(12)	0.00220(13)	0.00341(13)
O2	0.09983(7)	0.26818(6)	0.15527(7)	0.00564(7)	0.00567(15)	0.00528(15)	0.00750(16)	0.00047(12)	0.00437(13)	0.00230(12)
O3	0	0.63777(8)	0.25	0.00535(9)	0.0051(2)	0.0057(2)	0.0055(2)	0	0.00273(17)	0
H	0.110(2)	0.704(2)	0.294(2)	0.022(3)*						

**153 K**	*x*	*y*	*z*	U_{iso}*/U_{eq}	U^{11}	U^{22}	U^{33}	U^{12}	U^{13}	U^{23}
Co	0	0.5	0	0.00391(3)	0.00381(5)	0.00393(5)	0.00408(5)	-0.00008(3)	0.00195(3)	0.00027(3)
S	0	0.15498(3)	0.25	0.00364(4)	0.00310(7)	0.00347(7)	0.00437(7)	0	0.00180(5)	0
O1	0.17219(7)	0.04444(7)	0.40161(7)	0.00767(7)	0.00509(15)	0.00865(17)	0.00855(16)	0.00232(13)	0.00265(13)	0.00426(13)
O2	0.09929(7)	0.26795(6)	0.15505(7)	0.00672(7)	0.00699(16)	0.00600(16)	0.00904(16)	0.00071(12)	0.00532(13)	0.00279(12)
O3	0	0.63789(9)	0.25	0.00618(9)	0.0061(2)	0.0065(2)	0.0063(2)	0	0.00327(17)	0
H	0.110(2)	0.705(2)	0.293(2)	0.019(3)*						

**193 K**	*x*	*y*	*z*	U_{iso}*/U_{eq}	U^{11}	U^{22}	U^{33}	U^{12}	U^{13}	U^{23}
Co	0	0.5	0	0.00474(3)	0.00454(5)	0.00472(5)	0.00503(5)	-0.00008(3)	0.00235(3)	0.00034(3)
S	0	0.15468(2)	0.25	0.00432(4)	0.00356(7)	0.00404(7)	0.00533(7)	0	0.00210(5)	0
O1	0.17231(7)	0.04404(7)	0.40123(7)	0.00913(7)	0.00576(15)	0.01036(18)	0.01044(17)	0.00267(13)	0.00319(13)	0.00513(14)
O2	0.09866(7)	0.26764(6)	0.15475(7)	0.00793(7)	0.00812(16)	0.00695(16)	0.01093(16)	0.00101(12)	0.00634(13)	0.00341(13)
O3	0	0.63806(9)	0.25	0.00719(9)	0.0071(2)	0.0076(2)	0.0072(2)	0	0.00371(17)	0
H	0.110(2)	0.705(2)	0.294(2)	0.020(3)*						

**233 K**	*x*	*y*	*z*	U_{iso}*/U_{eq}	U^{11}	U^{22}	U^{33}	U^{12}	U^{13}	U^{23}
Co	0	0.5	0	0.00568(4)	0.00537(5)	0.00567(5)	0.00604(5)	-0.00009(3)	0.00275(4)	0.00038(3)
S	0	0.15433(3)	0.25	0.00510(4)	0.00412(7)	0.00475(7)	0.00634(7)	0	0.00242(5)	0
O1	0.17242(8)	0.04355(7)	0.40080(8)	0.01078(8)	0.00670(16)	0.01221(19)	0.01247(18)	0.00322(14)	0.00380(14)	0.00609(15)
O2	0.09802(8)	0.26722(6)	0.15443(7)	0.00931(8)	0.00939(17)	0.00810(17)	0.01282(18)	0.00123(13)	0.00722(15)	0.00394(14)
O3	0	0.63819(9)	0.25	0.00832(10)	0.0083(2)	0.0087(2)	0.0083(2)	0	0.00431(19)	0

H	0.103(2)	0.704(2)	0.294(2)	0.026(4)*						

**273 K**	*x*	*y*	*z*	U_{iso}*/U_{eq}	U^{11}	U^{22}	U^{33}	U^{12}	U^{13}	U^{23}
Co	0	0.5	0	0.00667(4)	0.00626(5)	0.00668(5)	0.00709(5)	-0.00011(3)	0.00322(4)	0.00044(3)
S	0	0.15394(3)	0.25	0.00590(4)	0.00478(7)	0.00550(7)	0.00729(7)	0	0.00277(5)	0
O1	0.17252(8)	0.04305(8)	0.40031(8)	0.01245(8)	0.00762(16)	0.0141(2)	0.01452(19)	0.00368(15)	0.00439(15)	0.00713(16)
O2	0.09729(8)	0.26680(7)	0.15409(8)	0.01077(8)	0.01087(17)	0.00944(17)	0.01480(19)	0.00154(14)	0.00840(15)	0.00458(14)
O3	0	0.63844(9)	0.25	0.00956(10)	0.0095(2)	0.0102(2)	0.0096(2)	0	0.00499(19)	0
H	0.102(2)	0.703(2)	0.294(2)	0.028(4)*						

**313 K**	*x*	*y*	*z*	U_{iso}*/U_{eq}	U^{11}	U^{22}	U^{33}	U^{12}	U^{13}	U^{23}
Co	0	0.5	0	0.00773(4)	0.00722(5)	0.00778(5)	0.00821(5)	-0.00010(3)	0.00373(4)	0.00052(3)
S	0	0.15349(3)	0.25	0.00676(4)	0.00542(7)	0.00632(7)	0.00835(7)	0	0.00315(5)	0
O1	0.17268(8)	0.04243(8)	0.39981(8)	0.01420(9)	0.00875(17)	0.0160(2)	0.0166(2)	0.00400(15)	0.00506(15)	0.00796(17)
O2	0.09640(8)	0.26637(7)	0.15370(8)	0.01236(8)	0.01246(18)	0.01089(18)	0.01701(19)	0.00190(14)	0.00970(16)	0.00524(15)
O3	0	0.63871(10)	0.25	0.01098(10)	0.0112(2)	0.0117(3)	0.0108(2)	0	0.0058(2)	0
H	0.101(2)	0.704(2)	0.295(2)	0.031(4)*						

$NiSO_4·H_2O$

**15 K**	*x*	*y*	*z*	U_{iso}*/U_{eq}	U^{11}	U^{22}	U^{33}	U^{12}	U^{13}	U^{23}
Ni	0	0.5	0	0.0077(2)	0.0073(3)	0.0069(3)	0.0093(3)	-0.00018(16)	0.0041(2)	-0.00008(14)
S	0	0.15772(12)	0.25	0.0078(3)	0.0075(5)	0.0072(5)	0.0092(5)	0	0.0041(4)	0
O1	0.1727(3)	0.0480(3)	0.4050(3)	0.0101(4)	0.0091(11)	0.0093(9)	0.0118(9)	-0.0003(8)	0.0047(8)	0.0013(7)
O2	0.1051(3)	0.2710(3)	0.1556(2)	0.0085(4)	0.0088(10)	0.0082(9)	0.0100(9)	-0.0003(7)	0.0056(7)	0.0009(7)
O3	0	0.6302(4)	0.25	0.0100(6)	0.0086(15)	0.0099(13)	0.0110(13)	0	0.0040(11)	0
H	0.113(6)	0.696(5)	0.296(5)	0.014(9)*						

**45 K**	*x*	*y*	*z*	U_{iso}*/U_{eq}	U^{11}	U^{22}	U^{33}	U^{12}	U^{13}	U^{23}
Ni	0	0.5	0	0.00160(19)	0.0011(3)	0.0023(3)	0.0017(3)	-0.00009(11)	0.00086(19)	-0.00006(11)
S	0	0.15779(9)	0.25	0.0018(2)	0.0010(4)	0.0022(4)	0.0023(4)	0	0.0009(3)	0
O1	0.1727(2)	0.0479(2)	0.4051(2)	0.0036(3)	0.0020(7)	0.0049(7)	0.0039(8)	0.0010(6)	0.0015(6)	0.0015(6)
O2	0.1052(2)	0.2713(2)	0.1554(2)	0.0035(3)	0.0028(7)	0.0043(7)	0.0038(7)	-0.0001(5)	0.0018(6)	0.0013(5)
O3	0	0.6308(3)	0.25	0.0035(4)	0.0014(10)	0.0044(10)	0.0036(11)	0	0.0003(9)	0
H	0.109(4)	0.691(4)	0.299(4)	0.012(7)*						

**75 K**	*x*	*y*	*z*	U_{iso}*/U_{eq}	U^{11}	U^{22}	U^{33}	U^{12}	U^{13}	U^{23}
Ni	0	0.5	0	0.0026(2)	0.0017(3)	0.0030(3)	0.0031(3)	-0.00013(12)	0.0011(2)	-0.00009(13)
S	0	0.15780(10)	0.25	0.0028(2)	0.0018(4)	0.0031(4)	0.0037(4)	0	0.0015(3)	0
O1	0.1724(2)	0.0479(2)	0.4049(2)	0.0047(4)	0.0031(8)	0.0049(7)	0.0054(8)	0.0008(6)	0.0015(7)	0.0012(6)
O2	0.1051(2)	0.2709(2)	0.1556(2)	0.0046(4)	0.0037(7)	0.0044(8)	0.0063(8)	0.0004(6)	0.0028(6)	0.0011(6)
O3	0	0.6304(3)	0.25	0.0046(5)	0.0024(11)	0.0055(11)	0.0061(12)	0	0.0022(10)	0
H	0.099(4)	0.698(4)	0.299(5)	0.024(9)*						

**113 K**	*x*	*y*	*z*	U_{iso}*/U_{eq}	U^{11}	U^{22}	U^{33}	U^{12}	U^{13}	U^{23}
Ni	0	0.5	0	0.00292(5)	0.00272(6)	0.00320(7)	0.00283(6)	-0.00010(4)	0.00127(5)	0.00010(3)
S	0	0.15763(4)	0.25	0.00284(5)	0.00228(9)	0.00297(10)	0.00321(9)	0	0.00121(7)	0
O1	0.17257(10)	0.04772(9)	0.40473(9)	0.00604(10)	0.0039(2)	0.0070(2)	0.0067(2)	0.00183(17)	0.00202(17)	0.00315(17)
O2	0.10507(10)	0.27108(8)	0.15552(9)	0.00506(9)	0.0051(2)	0.0047(2)	0.0067(2)	0.00031(16)	0.00378(17)	0.00181(16)
O3	0	0.63055(12)	0.25	0.00491(12)	0.0042(3)	0.0056(3)	0.0050(3)	0	0.0022(2)	0
H	0.109(3)	0.695(3)	0.294(3)	0.020(5)*						

**153 K**	*x*	*y*	*z*	U_{iso}*/U_{eq}	U^{11}	U^{22}	U^{33}	U^{12}	U^{13}	U^{23}
Ni	0	0.5	0	0.00357(5)	0.00321(6)	0.00392(7)	0.00349(6)	-0.00011(3)	0.00146(4)	0.00013(3)
S	0	0.15750(3)	0.25	0.00344(5)	0.00269(9)	0.00365(9)	0.00388(9)	0	0.00144(7)	0
O1	0.17263(10)	0.04756(9)	0.40448(9)	0.00712(10)	0.0044(2)	0.0084(2)	0.0080(2)	0.00214(17)	0.00232(17)	0.00384(17)
O2	0.10474(10)	0.27084(8)	0.15537(9)	0.00604(9)	0.0058(2)	0.0057(2)	0.0079(2)	0.00046(16)	0.00435(17)	0.00231(16)
O3	0	0.63067(11)	0.25	0.00555(12)	0.0050(3)	0.0062(3)	0.0054(3)	0	0.0024(2)	0
H	0.109(3)	0.695(3)	0.294(3)	0.018(5)*						

**193 K**	*x*	*y*	*z*	U_{iso}*/U_{eq}	U^{11}	U^{22}	U^{33}	U^{12}	U^{13}	U^{23}
Ni	0	0.5	0	0.00428(5)	0.00390(6)	0.00468(7)	0.00412(6)	-0.00014(4)	0.00173(5)	0.00016(3)
S	0	0.15727(3)	0.25	0.00404(5)	0.00314(9)	0.00423(10)	0.00453(9)	0	0.00159(7)	0
O1	0.17262(10)	0.04732(9)	0.40413(10)	0.00843(10)	0.0052(2)	0.0100(2)	0.0094(2)	0.00250(18)	0.00281(18)	0.00463(18)
O2	0.10428(10)	0.27059(8)	0.15519(9)	0.00703(9)	0.0067(2)	0.0066(2)	0.0094(2)	0.00068(16)	0.00512(18)	0.00264(16)
O3	0	0.63077(12)	0.25	0.00631(12)	0.0060(3)	0.0070(3)	0.0061(3)	0	0.0029(2)	0

H	0.109(3)	0.695(3)	0.294(3)	0.020(5)*						

**233 K**	*x*	*y*	*z*	U_{iso}*/U_{eq}	U^{11}	U^{22}	U^{33}	U^{12}	U^{13}	U^{23}
Ni	0	0.5	0	0.00492(5)	0.00440(7)	0.00540(7)	0.00470(7)	-0.00017(4)	0.00190(5)	0.00020(3)
S	0	0.15705(3)	0.25	0.00453(6)	0.00342(9)	0.00469(10)	0.00518(9)	0	0.00173(7)	0
O1	0.17277(10)	0.04702(10)	0.40390(10)	0.00962(11)	0.0057(2)	0.0114(3)	0.0109(2)	0.00282(19)	0.00316(18)	0.00550(19)
O2	0.10392(10)	0.27036(8)	0.15499(10)	0.00809(10)	0.0077(2)	0.0077(2)	0.0107(2)	0.00093(17)	0.00581(18)	0.00324(17)
O3	0	0.63099(12)	0.25	0.00704(12)	0.0064(3)	0.0078(3)	0.0069(3)	0	0.0031(2)	0
H	0.109(3)	0.695(3)	0.296(3)	0.018(4)*						

**273 K**	*x*	*y*	*z*	U_{iso}*/U_{eq}	U^{11}	U^{22}	U^{33}	U^{12}	U^{13}	U^{23}
Ni	0	0.5	0	0.00577(5)	0.00521(6)	0.00627(7)	0.00552(6)	-0.00020(3)	0.00223(4)	0.00023(3)
S	0	0.15681(3)	0.25	0.00527(5)	0.00403(9)	0.00542(9)	0.00597(9)	0	0.00201(7)	0
O1	0.17279(10)	0.04668(9)	0.40353(10)	0.01106(10)	0.0065(2)	0.0132(3)	0.0125(2)	0.00321(18)	0.00356(18)	0.00631(19)
O2	0.10339(10)	0.27006(8)	0.15477(9)	0.00926(10)	0.0089(2)	0.0086(2)	0.0122(2)	0.00120(16)	0.00657(18)	0.00377(17)
O3	0	0.63108(12)	0.25	0.00810(12)	0.0076(3)	0.0092(3)	0.0076(3)	0	0.0037(2)	0
H	0.108(3)	0.695(2)	0.294(3)	0.020(4)*						

**313 K**	*x*	*y*	*z*	U_{iso}*/U_{eq}	U^{11}	U^{22}	U^{33}	U^{12}	U^{13}	U^{23}
Ni	0	0.5	0	0.00653(5)	0.00586(6)	0.00709(7)	0.00627(6)	-0.00018(4)	0.00251(5)	0.00029(3)
S	0	0.15651(3)	0.25	0.00591(5)	0.00440(9)	0.00604(9)	0.00675(9)	0	0.00215(7)	0
O1	0.17290(10)	0.04625(10)	0.40317(10)	0.01253(11)	0.0073(2)	0.0148(3)	0.0144(2)	0.00359(19)	0.00413(19)	0.0072(2)
O2	0.10276(10)	0.26975(8)	0.15448(10)	0.01045(10)	0.0100(2)	0.0096(2)	0.0141(2)	0.00137(17)	0.00752(19)	0.00425(17)
O3	0	0.63132(12)	0.25	0.00908(13)	0.0085(3)	0.0103(3)	0.0087(3)	0	0.0043(2)	0
H	0.107(3)	0.696(3)	0.294(3)	0.023(5)*						

Table S3. Selected bond distances (Å) and angles (°) for kieserite-type compounds $M^{2+}SO_4{\cdot}H_2O$ (M^{2+} = Mg, Fe, Co, Ni) between 15 and 313 K. Respective room-temperature data for *M* = Mg and Co are given by Bechtold & Wildner (2016), for *M* = Fe by Talla & Wildner (2019), and for *M* = Ni by Talla *et al.* (2020). Note: S–O bond lengths are not corrected for thermal motion.

**$MgSO_4{\cdot}H_2O$**	*T* (K)	313	273	233	193	153	113	30	15
Mg–O1 (2×)		2.0232(6)	2.0230(6)	2.0229(6)	2.0222(5)	2.0222(5)	2.0222(5)	2.0170(21)	2.0223(12)
Mg–O2 (2×)		2.0401(5)	2.0405(5)	2.0409(5)	2.0407(5)	2.0413(5)	2.0410(5)	2.0403(22)	2.0405(13)
Mg–O3 (2×)		2.1726(4)	2.1697(4)	2.1671(4)	2.1640(4)	2.1619(4)	2.1607(4)	2.1596(17)	2.1583(9)
<Mg–O>		2.0786	2.0777	2.0770	2.0756	2.0751	2.0746	2.0723	2.0737
S–O1 (2×)		1.4621(5)	1.4628(5)	1.4634(5)	1.4640(5)	1.4643(5)	1.4647(5)	1.4647(22)	1.4647(13)
S–O2 (2×)		1.4809(5)	1.4820(5)	1.4832(5)	1.4837(5)	1.4843(4)	1.4849(4)	1.4826(22)	1.4838(12)
<S–O>		1.4715	1.4724	1.4733	1.4739	1.4743	1.4748	1.4737	1.4743
Mg–O1–S		140.38(4)	140.26(3)	140.12(3)	140.00(3)	139.91(3)	139.84(3)	139.96(14)	139.81(8)
Mg–O2–S		134.44(3)	134.16(3)	133.87(3)	133.63(3)	133.44(3)	133.32(3)	133.34(12)	133.30(7)
Mg–O3–Mg		123.32(4)	123.28(3)	123.27(3)	123.28(3)	123.28(3)	123.28(3)	123.26(16)	123.40(9)
O3···O2		2.7495(7)	2.7422(6)	2.7353(6)	2.7292(6)	2.7246(6)	2.7216(5)	2.7183(24)	2.7218(14)

**$FeSO_4{\cdot}H_2O$**	*T* (K)	313	273	233	193	153	113	75	60	45
Fe–O1 (2×)		2.1076(6)	2.1085(6)	2.1095(6)	2.1093(6)	2.1093(6)	2.1088(5)	2.1085(15)	2.1090(14)	2.1093(13)
Fe–O2 (2×)		2.0575(5)	2.0560(5)	2.0558(5)	2.0551(5)	2.0547(5)	2.0546(5)	2.0530(13)	2.0543(12)	2.0540(11)
Fe–O3 (2×)		2.2325(4)	2.2299(4)	2.2281(4)	2.2252(4)	2.2232(4)	2.2217(4)	2.2195(10)	2.2192(10)	2.2193(9)
<Fe–O>		2.1325	2.1315	2.1311	2.1299	2.1291	2.1284	2.1270	2.1275	2.1275
S–O1 (2×)		1.4647(5)	1.4646(5)	1.4649(5)	1.4652(5)	1.4656(5)	1.4657(5)	1.4645(15)	1.4630(14)	1.4635(13)
S–O2 (2×)		1.4856(5)	1.4868(5)	1.4879(5)	1.4887(5)	1.4892(5)	1.4901(5)	1.4908(13)	1.4903(12)	1.4896(11)
<S–O>		1.4752	1.4757	1.4764	1.4770	1.4774	1.4779	1.4777	1.4767	1.4766
Fe–O1–S		136.72(3)	136.55(3)	136.37(3)	136.22(3)	136.09(3)	136.04(3)	136.10(8)	136.08(8)	136.03(7)
Fe–O2–S		133.54(3)	133.30(3)	133.02(3)	132.79(3)	132.61(3)	132.45(3)	132.45(8)	132.42(8)	132.41(7)
Fe–O3–Fe		121.33(3)	121.32(3)	121.26(3)	121.28(3)	121.26(3)	121.27(3)	121.35(9)	121.36(9)	121.29(8)
O3···O2		2.7603(7)	2.7537(6)	2.7469(6)	2.7409(6)	2.7356(6)	2.7316(6)	2.7307(15)	2.7292(15)	2.7285(14)

**$CoSO_4 \cdot H_2O$** *T* (K)	313	273	233	193	153	113	30	15
Co–O1 (2×)	2.0567(6)	2.0564(6)	2.0556(6)	2.0555(6)	2.0555(6)	2.0557(5)	2.0559(21)	2.0540(22)
Co–O2 (2×)	2.0540(5)	2.0541(5)	2.0537(5)	2.0535(5)	2.0535(5)	2.0531(5)	2.0523(22)	2.0533(22)
Co–O3 (2×)	2.1804(4)	2.1778(4)	2.1753(4)	2.1736(4)	2.1720(4)	2.1704(4)	2.1633(17)	2.1654(17)
<Co–O>	2.0970	2.0961	2.0949	2.0942	2.0937	2.0931	2.0905	2.0909
S–O1 (2×)	1.4665(5)	1.4667(5)	1.4672(5)	1.4676(5)	1.4679(5)	1.4680(5)	1.4719(22)	1.4715(22)
S–O2 (2×)	1.4836(5)	1.4845(5)	1.4850(5)	1.4857(5)	1.4863(5)	1.4865(5)	1.4841(22)	1.4850(21)
<S–O>	1.4751	1.4756	1.4761	1.4767	1.4771	1.4773	1.4780	1.4783
Co–O1–S	136.98(3)	136.87(3)	136.76(3)	136.66(3)	136.57(3)	136.47(3)	136.29(14)	136.45(14)
Co–O2–S	132.83(3)	132.61(3)	132.44(3)	132.30(3)	132.15(3)	131.98(3)	131.88(13)	131.82(12)
Co–O3–S	122.25(3)	122.23(3)	122.22(3)	122.17(3)	122.15(3)	122.14(3)	122.35(16)	122.16(17)
O3···O2	2.7248(7)	2.7186(7)	2.7134(7)	2.7091(6)	2.7051(6)	2.7015(6)	2.7010(25)	2.6980(24)

**$Ni(SO_4) \cdot H_2O$** *T* (K)	313	273	233	193	153	113	75	45	15
Ni–O1 (2×)	2.0338(7)	2.0338(7)	2.0332(7)	2.0336(7)	2.0334(7)	2.0336(7)	2.0345(15)	2.0320(14)	2.0295(21)
Ni–O2 (2×)	2.0329(6)	2.0328(6)	2.0321(7)	2.0317(6)	2.0313(6)	2.0309(6)	2.0325(16)	2.0305(15)	2.0355(19)
Ni–O3 (2×)	2.1169(5)	2.1145(5)	2.1127(5)	2.1106(5)	2.1094(5)	2.1085(5)	2.1097(11)	2.1106(10)	2.1088(14)
<Ni–O>	2.0612	2.0604	2.0593	2.0586	2.0580	2.0577	2.0589	2.0577	2.0579
S–O1 (2×)	1.4654(7)	1.4654(6)	1.4659(7)	1.4657(7)	1.4668(6)	1.4672(7)	1.4670(16)	1.4687(15)	1.4685(21)
S–O2 (2×)	1.4865(6)	1.4869(6)	1.4874(6)	1.4872(6)	1.4879(6)	1.4888(6)	1.4879(16)	1.4905(15)	1.4877(18)
<S–O>	1.4760	1.47615	1.4767	1.47645	1.4774	1.4780	1.4775	1.4796	1.4781
Ni–O1–S	136.82(4)	136.75(4)	136.69(4)	136.63(4)	136.55(4)	136.47(4)	136.35(11)	136.42(10)	136.43(12)
Ni–O2–S	130.95(4)	130.79(4)	130.66(4)	130.57(4)	130.45(4)	130.38(4)	130.37(9)	130.35(9)	130.48(12)
Ni–O3–S	123.72(4)	123.73(4)	123.70(4)	123.72(4)	123.71(4)	123.72(4)	123.83(11)	123.60(10)	123.78(14)
O3···O2	2.6796(8)	2.6760(8)	2.6725(8)	2.6706(8)	2.6681(8)	2.6667(8)	2.6662(17)	2.6640(17)	2.6630(22)
